# Interferon and B-cell Signatures Inform Precision Medicine in Lupus Nephritis

**DOI:** 10.1016/j.ekir.2024.03.014

**Published:** 2024-03-13

**Authors:** Ioannis Parodis, Julius Lindblom, Daniel Toro-Domínguez, Lorenzo Beretta, Maria O. Borghi, Jessica Castillo, Elena Carnero-Montoro, Yvonne Enman, Chandra Mohan, Marta E. Alarcón-Riquelme, Guillermo Barturen, Dionysis Nikolopoulos, PRECISESADS Clinical Consortium, Lorenzo Beretta, Barbara Vigone, Jacques-Olivier Pers, Alain Saraux, Valérie Devauchelle-Pensec, Divi Cornec, Sandrine Jousse-Joulin, Bernard Lauwerys, Julie Ducreux, Anne-Lise Maudoux, Carlos Vasconcelos, Ana Tavares, Esmeralda Neves, Raquel Faria, Mariana Brandão, Ana Campar, António Marinho, Fátima Farinha, Isabel Almeida, Miguel Angel Gonzalez-Gay Mantecón, Ricardo Blanco Alonso, Alfonso Corrales Martínez, Ricard Cervera, Ignasi Rodríguez-Pintó, Gerard Espinosa, Rik Lories, Ellen De Langhe, Nicolas Hunzelmann, Doreen Belz, Torsten Witte, Niklas Baerlecken, Georg Stummvoll, Michael Zauner, Michaela Lehner, Eduardo Collantes, Rafaela Ortega-Castro, Ma Angeles Aguirre-Zamorano, Alejandro Escudero-Contreras, Ma Carmen Castro-Villegas, Norberto Ortego, María Concepción Fernández Roldán, Enrique Raya, Inmaculada Jiménez Moleón, Enrique de Ramon, Isabel Díaz Quintero, Pier Luigi Meroni, Maria Gerosa, Tommaso Schioppo, Carolina Artusi, Carlo Chizzolini, Aleksandra Zuber, Donatienne Wynar, Laszló Kovács, Attila Balog, Magdolna Deák, Márta Bocskai, Sonja Dulic, Gabriella Kádár, Falk Hiepe, Velia Gerl, Silvia Thiel, Manuel Rodriguez Maresca, Antonio López-Berrio, Rocío Aguilar-Quesada, Héctor Navarro-Linares

**Affiliations:** 1 Division of Rheumatology, Department of Medicine Solna, Karolinska Institutet, Stockholm, Sweden; 2 Department of Gastroenterology, Dermatology, and Rheumatology, Karolinska University Hospital, Stockholm, Sweden; 3 Department of Rheumatology, Faculty of Medicine and Health, Örebro University, Örebro, Sweden; 4 GENYO, Centre for Genomics and Oncological Research: Pfizer, University of Granada / Andalusian Regional Government, Granada, Spain, Medical Genomics, Granada, Spain; 5 Referral Center for Systemic Autoimmune Diseases, Fondazione IRCCS Ca’ Granda Ospedale Maggiore Policlinico di Milano, Italy; 6 Department of Clinical Sciences and Community Health, Università Degli Studi di Milano, Milan, Italy; 7 IRCCS, Istituto Auxologico Italiano, Milan, Italy; 8 Department of Biomedical Engineering, University of Houston, Houston, Texas, USA; 9 Department of Environmental Medicine, Karolinska Institute, Stockholm, Sweden; 10 Department of Genetics, Faculty of Sciences, University of Granada, Granada, Spain

**Keywords:** lupus nephritis, systemic lupus erythematosus, precision medicine, druggability, biologics, transcriptome, trancriptomics

## Abstract

**Introduction:**

Current therapeutic management of lupus nephritis (LN) fails to induce long-term remission in over 50% of patients, highlighting the urgent need for additional options.

**Methods:**

We analyzed differentially expressed genes (DEGs) in peripheral blood from patients with active LN (*n* = 41) and active nonrenal lupus (*n* = 62) versus healthy controls (HCs) (*n* = 497) from the European PRECISESADS project (NTC02890121), and dysregulated gene modules in a discovery (*n* = 26) and a replication (*n* = 15) set of active LN cases.

**Results:**

Replicated gene modules qualified for correlation analyses with serologic markers, and regulatory network and druggability analysis. Unsupervised coexpression network analysis revealed 20 dysregulated gene modules and stratified the active LN population into 3 distinct subgroups. These subgroups were characterized by low, intermediate, and high interferon (IFN) signatures, with differential dysregulation of the “B cell” and “plasma cells/Ig” modules. Drugs annotated to the IFN network included CC-motif chemokine receptor 1 (CCR1) inhibitors, programmed death-ligand 1 (PD-L1) inhibitors, and irinotecan; whereas the anti-CD38 daratumumab and proteasome inhibitor bortezomib showed potential for counteracting the “plasma cells/Ig” signature. *In silico* analysis demonstrated the low-IFN subgroup to benefit from calcineurin inhibition and the intermediate-IFN subgroup from B-cell targeted therapies. High-IFN patients exhibited greater anticipated response to anifrolumab whereas daratumumab appeared beneficial to the intermediate-IFN and high-IFN subgroups.

**Conclusion:**

IFN upregulation and B and plasma cell gene dysregulation patterns revealed 3 subgroups of LN, which may not necessarily represent distinct disease phenotypes but rather phases of the inflammatory processes during a renal flare, providing a conceptual framework for precision medicine in LN.

## Graphical abstract

**Figure:**
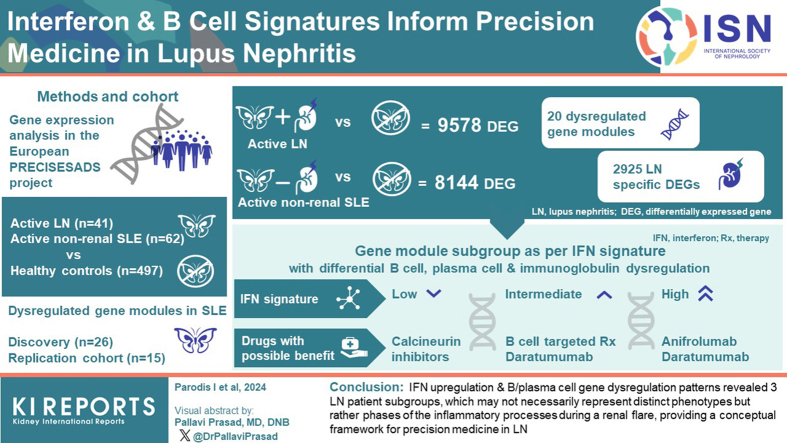

LN represents a significant and severe complication of systemic lupus erythematosus (SLE), affecting up to 50% of patients.^1^^,^^2^ Despite the advancements achieved with the introduction of broad immunosuppressive and targeted therapies, patients with LN continue to exhibit unacceptable rates of end-stage kidney disease and mortality.^3^ Although these interventions have altered the evolution of the disease,^4^ there remains a pressing need for further improvement in the management of LN, with the goal of reducing the burden of complications and enhancing long-term survival.

The current therapeutic approach for LN consists of an initial phase of therapy utilizing high-dose glucocorticoids (GCs) along with low-dose i.v. cyclophosphamide or oral mycophenolate, followed by a phase of less intense therapy intended to maintain remission.^5^ However, this treatment strategy falls short in achieving long-term remission in more than 50% of LN cases. Notably, clinically quiescent disease often comes along with residual kidney inflammation, and patients frequently experience relapses, chronic exposure to GCs, and drug-induced toxicity.^2^^,^^6^^,^^7^

After facing setbacks for many years, 2 phase 3 clinical trials evaluating belimumab and voclosporin as add-on therapies to conventional agents, particularly mycophenolate, revealed potentiality for better renal response and facilitated quick tapering of GCs.^8^^,^^9^ However, long-term outcomes and the benefit-to-risk ratio of these drugs still need to be determined in real-world settings. Furthermore, it remains uncertain whether these agents will become the first-line choice for all patients with LN from the outset or if their use will be prioritized for specific cases.

To date, many other agents failed in multiple SLE clinical trials owing to the wide range and heterogeneity of immune pathways that are involved in SLE and LN pathogenesis.^10^ Indeed, in our previous work, we showed that different pathogenetic mechanisms may underlie patients with lupus with the same organ involvement.^11^ In this regard, growing evidence from omics studies points toward precision medicine potentiality and tailored treatment strategies.^12^

The aim of this study was to investigate the LN transcriptome in-depth to gain insights into the underlying molecular mechanisms and to identify new potential drug targets for LN. We addressed the latter by following 2 separate druggability pipelines, including *in silico* prediction of response to currently existing targeted therapies, though the majority are not trialed or approved, paving the way toward precision medicine in LN.

## Methods

### Study Population

Peripheral blood samples and clinical data were collected from patients with SLE (*n* = 350) who all met the revised American College of Rheumatology criteria for SLE,^13^ as well as from 497 healthy controls (HCs), within the frame of the 5-year European PRECISESADS project (NTC02890121).^14^

The full list of inclusion and exclusion criteria can be found in Supplementary Table S1. Active LN was defined as proteinuria of ≥0.5 g/d in patients with a history of biopsy-proven LN (*n* = 41). Active nonrenal SLE was defined as no history of renal involvement and no history of or current proteinuria in patients with a score of ≥4 in the clinical version of SLE Disease Activity Index 2000 (SLEDAI-2K) and 0 score in the renal domain of SLEDAI-2K (*n* = 62).^15^ Patients with LN and nonrenal SLE were matched to HCs based on age and sex at a 1:5 ratio, resulting in 205 HCs and 310 HCs, respectively.

Before inclusion, all patients and HCs signed informed consent, and the study had received approval from local ethics review boards (Supplementary Material, List of local investigators). The investigation of the current study was approved by the Swedish Ethical Review Authority (reference: 2022-03907-01).

### Sample Data

Genome-wide peripheral whole-blood RNA-sequencing was performed using Illumina assays (Illumina Inc., San Diego, CA), as described elsewhere.^14^ Following the removal of genes with low counts, raw counts of the remaining genes were transformed into log_2_ counts per million. In the case of duplicated gene symbols, the mean count was used. Serum levels of a wide range of selected cytokines and autoantibodies were determined, as described elsewhere.^14^ Initially, a total of 88 cytokines were analyzed in a subset of patients and HCs using Luminex xMAP Technology from the Luminex Corporation (Austin, TX). Subsequently, a customized panel from R&D Systems (Luminex assay, Luminex Corporation, Austin, TX) was used to measure a subset of 14 cytokines, whereas a quantitative sandwich enzyme immunoassay from Biorad Laboratories Inc. (Hercules, CA) was used to analyze 6 additional cytokines. Autoantibody levels were measured using an automated chemiluminescent immunoassay (IDS-iSYS, Immunodiagnostic Systems Holdings Ltd., East Boldon, UK), a turbidimetric immunoassay (SPAPLUS analyzer, The Binding Site Group Ltd., Birmingham, UK), and an enzyme-linked immunosorbent assay kit from EUROIMMUN Medizinische Labordiagnostika AG (Lübeck, Germany).

### Statistical and Bioinformatic Analysis

To determine LN-specific enriched pathways, we performed DEG analysis with the limma R package,^16^ using voom transformation and adjusting for age, sex, sequencing batch, and RNA integrity number. First, DEG analysis was performed in patients with active LN (*n* = 41) versus HC (*n* = 497). A subsequent DEG analysis was performed in patients with active nonrenal SLE (*n* = 62) versus HCs (*n* = 497). Significant DEGs from both analyses were visualized with the VennDiagram R package.^17^ Pathway enrichment analysis was performed by means of overrepresentation analysis with the ClusterProfiler^18^ and ReactomePA^19^ R packages using LN-specific genes to yield enriched biological process and molecular function gene ontology terms,^20^ and Reactome pathways.^21^ A false discovery rate <0.05 (Benjamini-Hochberg) was deemed statistically significant.

Dysregulation of gene modules was performed using weighted gene coexpression network analysis (WGCNA), functional annotation based on gene expression data from Chaussabel *et al.*^22^ and Li *et al.*,^23^ and by calculating mean *z*-scores for each patient with LN compared with HCs, as described previously.^11^^,^^24^ For weighted gene coexpression network analysis, the patients with LN were analyzed separately in a discovery set using data from the PRECISESADS 1 patient population (*n* = 26) and a replication set using data from a separate and independent patient population, that is, PRECISESADS 2 (*n* = 15). The 2 cohorts were independent; however, they only differed in that PRECISESADS 1 was completed and used for the first phase of the project, that is, first data generation set, whereas PRECISESADS 2 formed the continuation of the project. A similar weighted gene coexpression network analysis was performed in a discovery set using data from the PRECISESADS 1 active nonrenal SLE patient population (*n* = 43) and a replication set using data from the PRECISESADS 2 active nonrenal SLE patient population (*n* = 19). Replicated dysregulated gene modules were clustered based on their dysregulation scores using hierarchical clustering with the Ward method and visualized with the pheatmap R package.^25^ Gene modules with a mean |*z*-score| >1 in at least 1 patient subgroup underwent an additional round of clustering and the genes in these gene modules were imputed in iRegulon^26^ through Cytoscape^27^ to generate signaling molecule networks and identify their chief regulators. Genes included in one of the 3 most enriched signaling molecule networks were then analyzed for druggability using the Drug Gene Interaction database with the rDGIdb R package.^28^ We subsequently annotated upregulated and downregulated genes in each patient subgroup with a mean |*z*-score| >1 in the respective gene module, along with their respective inhibitors or stimulators found in the most enriched signaling molecule network from the aforementioned analysis.

Gene expression data were further subjected to druggability analysis with the hipathia R package,^29^ following the instructions provided by its authors. The gene expression data of patients with LN were normalized to that of matched HCs, and response scores for modulating selected drug targets for each patient were calculated as the absolute change of gene expression before and after the target inhibition, using a multiplication factor of 0.1 to simulate inhibition. Drug targets tested were selected based on the preceding druggability analysis and expert opinion. An anticipated favorable response to a drug for each patient with LN was defined as a response score equal to or greater than the mean response scores of all patients.

Comparisons of unrelated continuous data were performed using the Mann-Whitney *U* test. Associations between unrelated binomial variables were investigated using the Pearson’s chi-square (*χ*^2^) or the Fisher’s exact test, as appropriate. Correlation analyses were conducted using Pearson correlation coefficients for functional annotation of gene modules or Spearman’s rank correlation coefficients for analysis of dysregulation of gene modules in relation to serum levels of selected cytokines. Differences yielding a *P*-value < 0.05 were considered statistically significant. All analyses were conducted using the R software, version 4.3.1 (R Foundation for Statistical Computing, Vienna, Austria). All analyses were conducted in accordance with Strengthening the Reporting of OBservational studies in Epidemiology (STROBE; Supplementary Table S2).

## Results

Demographics and clinical data of patients and HCs are presented in Table 1. The SLEDAI-2K scores did not differ between patients with active LN and active nonrenal SLE (mean [standard deviation]: 12.2 [7.5] vs. 11.2 [6.4]; *P* = 0.487).

**Table 1:** Characteristics of patients with active LN, active nonrenal SLE, and healthy controls from the PRECISESADS study population

Characterisitcs	Discovery set: active LN (*n* = 25)	Replication set: active LN (*n* = 16)	Comparators	
			Active nonrenal SLE (*n* = 62)	HC (*n* = 205)
Demographics				
Age (yr), mean ± SD	42.1 ± 13.6	46.7 ± 14.6	46.8 ± 13.3	43.8 ± 13.3
Female sex, *n* (%)	22 (88.0)	14 (87.5)	60 (96.8)	180 (87.8)
European origin, *n* (%)	25 (100)	16 (100)	62 (100)	205 (100)
Clinical data, mean ± SD				
Disease duration (yr)	15.1 ± 9.9	18.7 ± 7.4	13.2 ± 10.4	N/A
SLEDAI-2K score	12.8 ± 8.1	11.3 ± 6.6	11.2 ± 6.4	N/A
Serologic profile				
Anti-dsDNA (U/ml); median (IQR)	82.8 (9.2–191.5)	29.4 (15.5–50.6); *n =* 6	8.6 (0.0–47.0); *n =* 48	N/A
Anti-dsDNA (+); *n* (%)	16 (64.0)	2 (33.3); *n =* 6	13 (27.1); *n =* 48	N/A
Anti-Sm (U/ml); median (IQR)	0.0 (0.0–0.0); *n =* 20	0.0 (0.0–0.0); *n =* 5	0.0 (0.0–0.0); *n =* 45	N/A
Anti-Sm (+); *n* (%)	2 (10.0); *n =* 20	0 (0); *n =* 5	1 (2.2); *n =* 45	N/A
Anti-β_2_GPI IgG (U/ml); median (IQR)	0.0 (0.0–1.0)	0.1 (0.0–123.2); *n =* 6	0.0 (0.0–0.0); *n =* 47	N/A
Anti-β_2_GPI IgG (+); *n* (%)	3 (12.0)	2 (33.3); *n =* 6	5 (10.6); *n =* 47	N/A
Anti-β_2_GPI IgM (U/ml); median (IQR)	0.0 (0.0–0.1)	0.0 (0.0–1.6); *n =* 6	0.0 (0.0–0.8); *n =* 47	N/A
Anti-β_2_GPI IgM (+); *n* (%)	1 (4.0)	0 (0.0); *n =* 6	5 (10.6); *n =* 47	N/A
aCL IgG (U/ml); median (IQR)	0.0 (0.0–1.8)	0.0 (0.0–36.7); *n =* 6	0.0 (0.0–0.6); *n =* 48	N/A
aCL IgG (+); *n* (%)	2 (8.0)	2 (33.3); *n =* 6	6 (12.5); *n =* 48	N/A
aCL IgM (U/ml); median (IQR)	0.0 (0.0–0.3)	0.0 (0.0–0.0); *n =* 6	0.0 (0.0–1.6); *n =* 48	N/A
aCL IgM (+); *n* (%)	0 (0.0)	0 (0.0); *n =* 6	5 (10.4); *n =* 48	N/A
Low C3c (g/l); median (IQR)	1.03 (0.7–1.3)	1.1 (0.8–1.4); *n =* 6	1.0 (0.6–1.3); *n =* 48	N/A
Low C3c; *n* (%)	8 (32.0)	2 (33.3); *n =* 6	22 (45.8); *n =* 48	N/A
Low C4 (g/l); median (IQR)	0.2 (0.1–0.3)	0.2 (0.1–0.3); *n =* 6	0.2 (0.1–0.3); *n =* 48	N/A
Low C4; *n* (%)	8 (32.0)	1 (16.7); *n =* 6	19 (39.6); *n =* 48	N/A
Medications (current use)				
Prednisone equivalent dose (mg/d); mean ± SD	4.1 ± 3.9; *n =* 17	3.6 ± 3.1; *n =* 12	3.7 ± 3.6; *n =* 56	N/A
Antimalarial agents; *n* (%)	19 (76.0)	13 (81.2)	50 (80.6)	N/A
Immunosuppressants; *n* (%)				
Azathioprine	9 (42.9); *n =* 21	2 (18.2); *n =* 11	7 (11.9); *n =* 59	N/A
Calcineurin inhibitors	0 (0.0); *n* = 18	1 (8.3); *n =* 12	0 (0.0); *n =* 59	N/A
Leflunomide	0 (0.0); *n* = 21	0 (0.0); *n =* 11	1 (1.7); *n =* 59	N/A
Methotrexate	3 (14.3); *n* = 21	1 (9.1); *n =* 11	11 (18.6); *n =* 59	N/A
Mycophenolic acid	8 (38.1); *n* = 21	4 (33.3); *n =* 12	3 (5.1); *n =* 59	N/A

aCL, antibodies against cardiolipin; anti-β_2_GPI, antibodies against β_2_-glycoprotein I; anti-dsDNA, antibodies against double-stranded DNA; anti-Sm, antibodies against Smith; C3c, complement component 3c; C4, complement component 4; HC, healthy controls; IQR, interquartile range; LN, lupus nephritis; N/A, not applicable; SD, standard deviation; SLE, systemic lupus erythematosus; SLEDAI-2K, Systemic Lupus Erythematosus Disease Activity Index 2000.

Data are presented as the number (percentage) or mean ± SD. In case of nonnormal distributions, the median (IQR) is indicated. In case of missing values, the total number of patients with available data is indicated.

### Transcriptomic Aberrancies in LN

**Figure 1:**
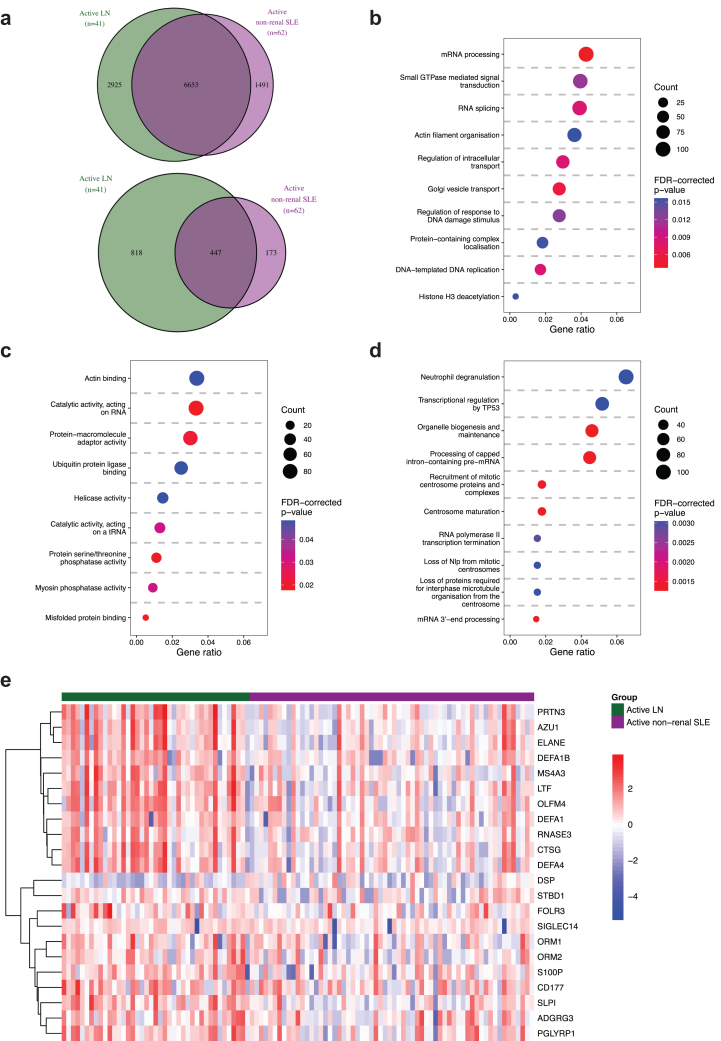
DEGs in patients with active LN and active nonrenal SLE versus HC. (a) The Venn diagrams show DEGs (top) and the subset of DEGs that exceeded the |log_2_ FC| > 0.58 threshold (bottom) in patients with active LN versus HCs (in green), and in patients with active nonrenal SLE versus HC (in purple). The most enriched (b) BP GO terms, (c) MF GO terms, and (d) Reactome pathways from overrepresentation analysis are plotted, based on LN-specific DEGs in patients with active LN versus HCs (panel A, *n* = 2925). The size of the dots represents the gene count, and the gene ratio at the bottom of each dot plot represents the ratio between the gene count and the total number of DEGs included in the GO or Reactome library. The color of the dots corresponds to the FDR-corrected *P*-value from the pathway enrichment analysis. (e) The heatmap shows gene expression patterns in patients with active LN (green) or active nonrenal SLE (purple) from the Reactome “neutrophil degranulation” pathway. Only DEGs that exceeded the |log_2_ FC| > 0.58 threshold in the DEG analysis in patients with active LN versus HCs are included in the heatmap. Columns denote SLE patients, and rows denote LN-specific DEGs, clustered using hierarchical clustering with the Ward method. BP, biological process; CPM, counts per million; DEGs, differentially expressed genes; FC, fold change; FDR, false discovery rate; GO, gene ontology; HCs, healthy controls; LN, lupus nephritis; MF, molecular function; SLE, systemic lupus erythematosus.

We found 9578 DEGs in active LN versus HCs and 8144 DEGs in active nonrenal SLE versus HC (Supplementary Material, Sheets 1 and 2). Patients with active LN and active nonrenal SLE shared 6653 DEGs, whereas 2925 DEGs were exclusively dysregulated in the LN group, denoting an LN-specific signature (Figure 1a). Among the latter, 818 DEGs displayed a |log_2_ fold change| >0.58, that is, fold change <0.66 for downregulation and >1.5 for upregulation (Figure 1a). The LN signature comprised DEGs associated with neutrophilic degranulation, transcription regulation by TP53, and DNA damage response gene ontology pathways (Figure 1b–d and Supplementary Material, Sheets 3–5). We identified *ELANE*, *CTSG*, *DEFA1*, and *DEFA4* as putative upstream regulators of the LN signature, implicating a role for neutrophils as key mediators in LN (Figure 1e and Supplementary Material, Sheet 5).

### Dysregulated Gene Modules and Molecular Subgroups in LN

**Figure 2:**
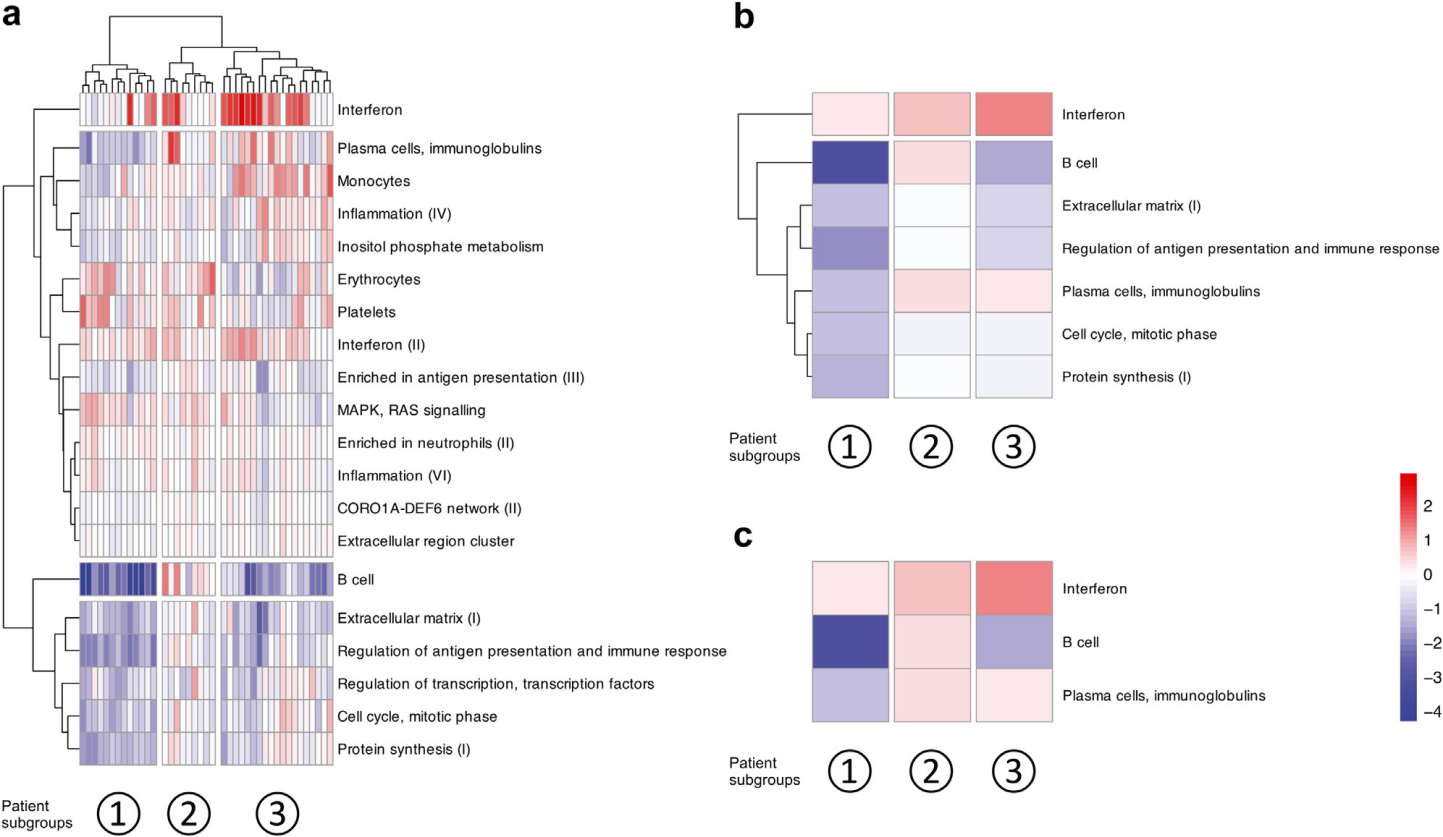
Dysregulated gene modules in patients with active LN. (a) The heatmap shows replicated gene modules and their dysregulation in relation to the gene expression of HCs, as measured by the *z*-score, in patients with LN. Columns denote patients with LN, and rows denote gene modules, clustered using hierarchical clustering with the Ward method. (b) The heatmap displays gene modules with a mean |*z*-score| >1 in at least 1 patient subgroup, represented in each column. (c) The heatmap displays the most discriminative gene modules, categorizing the patients into a lo-IFN, im-IFN, and high hi-IFN subgroup. Red and blue colors denote higher and lower *z*-scores compared to the HC, respectively. CORO1A-DEF6, coronin 1A-differentially expressed in FDCP 6 homolog; HCs, healthy controls; hi, high; IFN, interferon; im, intermediate; lo, low; LN, lupus nephritis; MAPK, mitogen-activated protein kinase.

WGCNA analysis identified 20 replicated gene modules among 41 patients with LN (Supplementary Material, Sheets 6–8). Of the 20 replicated gene modules identified in the active LN group, 5 were also identified in the active nonrenal SLE group, and 3 were contiguous (Supplementary Material, Sheet 6). These 8 gene modules, common to both the active LN and active nonrenal SLE group, denoted gene modules related to systemic SLE activity, whereas the remaining 12 were classified as LN-specific gene modules. The 20 dysregulated gene modules were then organized into 4 main clusters, including a distinct “IFN” cluster and a “B cell” gene module cluster. In the same analysis, the patients with LN were categorized into 3 distinct subgroups (Figure 2a). Subsequent clustering of the replicated gene modules with prominent dysregulation revealed 2 main gene module clusters, further highlighting the importance of the distinct IFN gene module cluster (Figure 2b). Across the 3 patient subgroups, we observed a differential degree of upregulation of the IFN gene module, resulting in the categorization of patients into a low (lo)-IFN, an intermediate (im)-IFN, and a high (hi)-IFN patient subgroup. Notably, the lo-IFN patient subgroup exhibited substantial downregulation of both the “B cell” and “plasma cells, Ig” gene modules. In contrast, the im-IFN subgroup showed upregulation of both the “B cell” and “plasma cells, Ig” gene modules. The hi-IFN subgroup was characterized by downregulation of the “B cell” gene module and upregulation of the “plasma cells, Ig” gene module (Figure 2c). The patient subgroups did not differ in renal disease activity, as measured by the renal components of SLEDAI-2K, that is, renal SLEDAI-2K (Supplementary Table S3).

### Cytokine and Autoantibody Profiles in Relation to Dysregulated Gene Modules in LN

**Figure 3:**
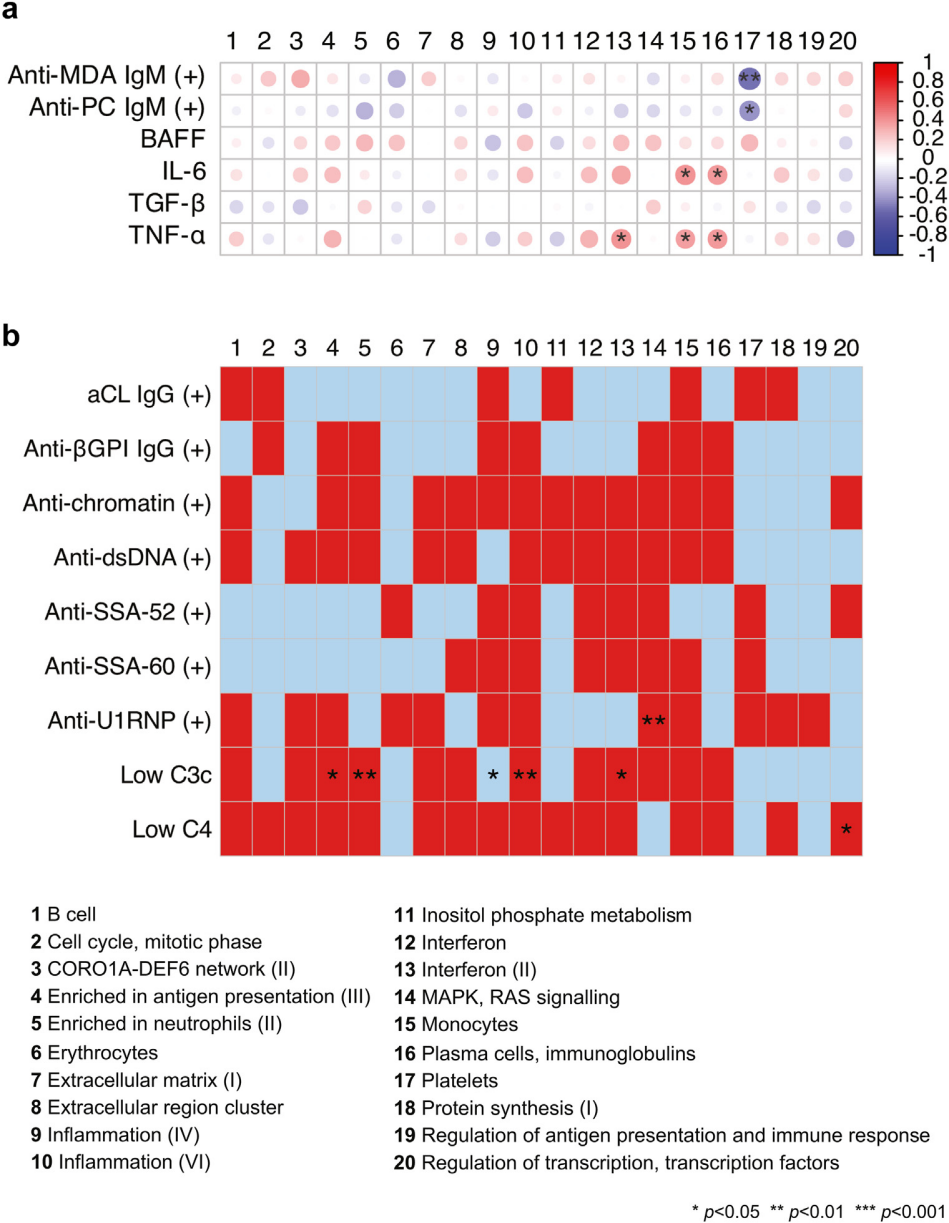
Dysregulated gene modules in relation to serologic markers in patients with active lupus nephritis. (a) The correlation heatmap shows Spearman’s rank correlation coefficients for correlations between levels of selected autoantibodies or cytokines and dysregulation of gene modules as measured by the *z*-score. (b) Dysregulation of gene modules in relation to autoantibody positivity or low levels of C3c or C4. The group without autoantibody positivity or low levels of C3c or C4 for each comparison was considered the reference group. Red and blue colors denote higher and lower *z*-scores compared with the reference group, respectively. *P*-values are derived from Mann-Whitney *U* tests. Asterisks denote statistically significant correlations or differences. aCL, anticardiolipin; BAFF, B-cell activating factor belonging to the tumor necrosis factor family; βGPI, β2 glycoprotein I; C3c, complement component 3c; C4, complement component 4; CORO1A-DEF6, coronin 1A - differentially expressed in FDCP 6 homolog; IL-6, interleukin 6; MAPK, mitogen-activated protein kinase; MDA, malondialdehyde; PC, phosphorylcholine; TGF-β, transforming growth factor β; TNF-α, transforming growth factor α.

The dysregulation of the “platelets” gene module was negatively correlated with antiphosphorylcholine and antimalondialdehyde IgM antibody levels in patients with active LN (Figure 3a; Supplementary Tables S4–S23). In contrast, the dysregulation scores of the “monocytes” and “plasma cells, Ig” gene modules showed a positive correlation with serum IL-6 and TNF-α levels, respectively. Moreover, the dysregulation of the “interferon (II)” gene module was positively correlated with serum TNF-α levels. The dysregulation score of several gene modules, including the IFN (II) gene module, was higher in patients with low versus normal or high levels of C3c (Figure 3b; Supplementary Tables S24–S32).

### Druggability Potentiality in LN

**Figure 4:**
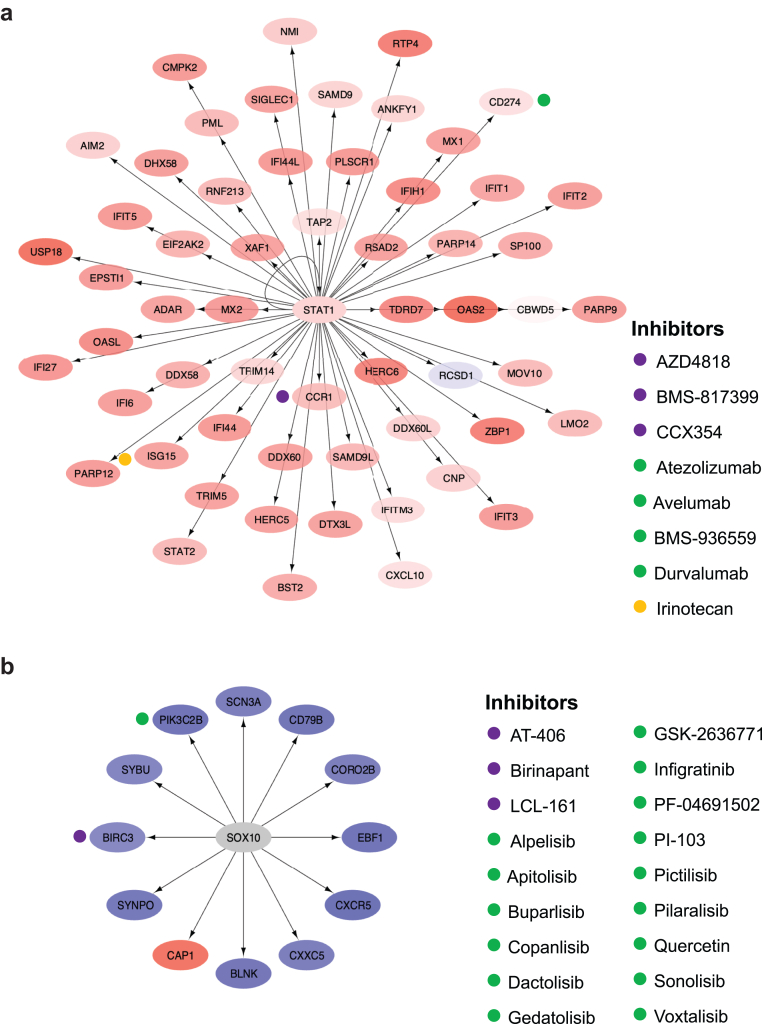
The *STAT1* and *SOX10* signaling molecule networks and annotated drug targets in patients with active lupus nephritis. (a) Genes in the interferon gene module were imputed in iRegulon through Cytoscape to generate signaling molecule networks and identify their chief regulators. One of the most enriched signaling molecule networks, based on normalized enrichment score, is plotted, with the chief regulator *STAT1* in the central node. The color of the nodes ranges from light blue (downregulated genes) to increasing intensities of red (upregulated genes) based on the gene dysregulation (*z*-scores) in the hi-IFN patient subgroup. (b) One of the most enriched signaling molecule networks, based on genes in the B-cell gene module, is plotted, with the chief regulator *SOX10* in the central node. The color of the nodes ranges from light blue (downregulated genes) to increasing intensities of red (upregulated genes) based on the gene dysregulation (*z*-scores) in the lo-IFN and hi-IFN patient subgroups. Hi, high; IFN, interferon; lo; low; *STAT1*: signal transducer and activator of transcription 1.

**Figure 5:**
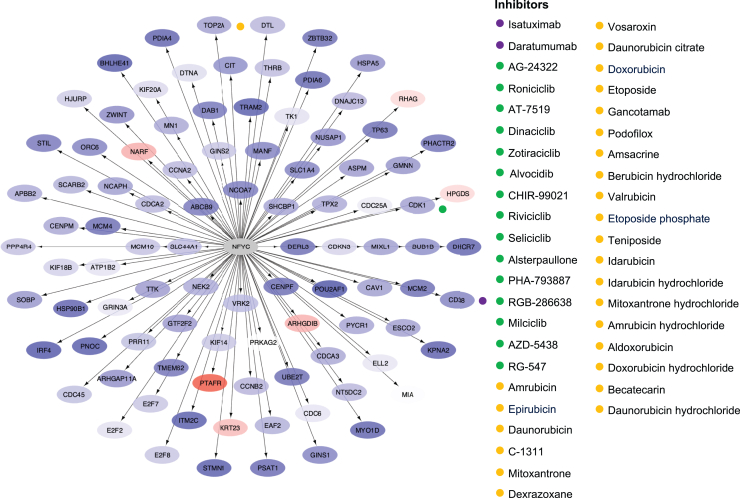
The NFYC signaling molecule network and annotated drug targets in patients with active lupus nephritis. Genes in the plasma cells, Ig gene module were imputed in iRegulon through Cytoscape to generate signaling molecule networks and identify their chief regulators. One of the most enriched signaling molecule networks, based on normalized enrichment score, is plotted, with the chief regulator NFYC in the central node. The color of the nodes ranges from light blue (downregulated genes) to increasing intensities of red (upregulated genes) based on the gene dysregulation (*z*-scores) in the lo-IFN patient subgroup. Inhibiting drugs and their upregulated targets are indicated by colored dots. IFN, interferon; lo, low; NFYC, nuclear transcription factor Y subunit gamma.

The chief regulators and motifs of the most enriched signaling molecule networks that were identified from genes in the replicated gene modules with prominent dysregulation are presented in Supplementary Table S33. Signal transducer and activator of transcription 1 was identified as the chief regulator in the most enriched signaling molecule network, based on genes in the IFN gene module, as shown for the hi-IFN patient subgroup in Figure 4a. Drugs annotated to genes in this network included CCR1 inhibitors; PD-L1 inhibitors; and irinotecan, which inhibits the expression of IFN-stimulated gene 15 (i.e., *ISG15*). In the SRY-box transcription factor 10-regulated gene network, based on the B-cell gene module that showed prominent dysregulation in the lo-IFN and hi-IFN patient subgroups, annotated drugs included cellular inhibitors of apoptosis 1/2 and phosphoinositide 3-kinases (PI3K) (Figure 4b). Analysis of the downregulated plasma cells, Ig gene module in the lo-IFN subgroup yielded a nuclear transcription factor Y subunit gamma-regulated network, which showed druggability potential for targeting *CD38* with the anti-CD38 monoclonal daratumumab (Figure 5). The results from the analysis of signaling molecule networks with druggability potentiality are detailed in the Supplementary Material Sheet 9 and Supplementary Figures S1 to S4.

**Figure 6:**
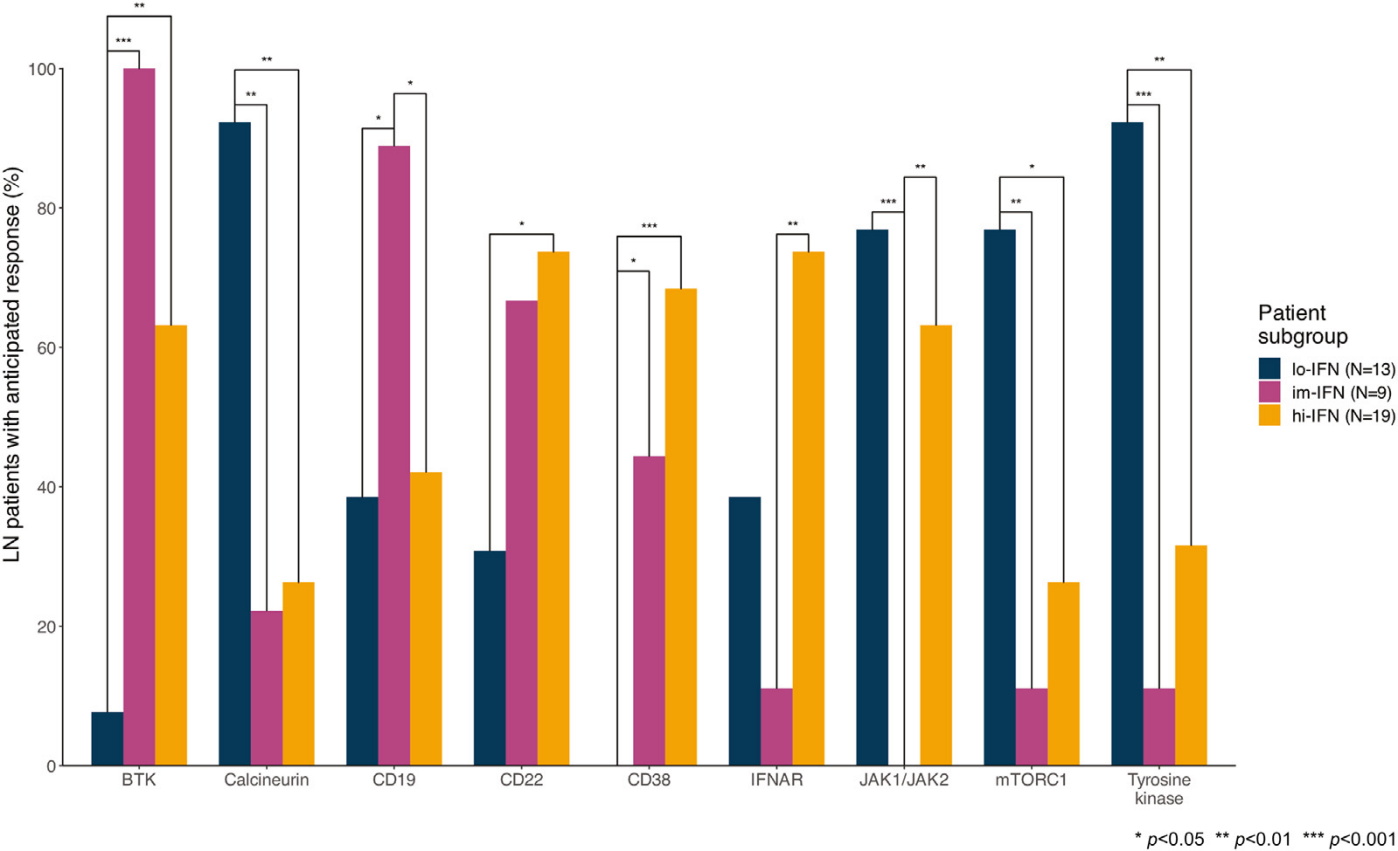
Anticipated response to inhibition of selected drug targets in patients with active lupus nephritis (LN). Bars depict proportions of patients with an anticipated benefit from inhibition of selected drug targets across LN patient subgroups. BTK, Bruton’s tyrosine kinase; hi, high; IFN, interferon; IFNAR, interferon-α/β receptor; im, intermediate; JAK, Janus kinase; LN, lupus nephritis; lo, low; mTORC1, mammalian target of rapamycin complex 1.

**Figure 7:**
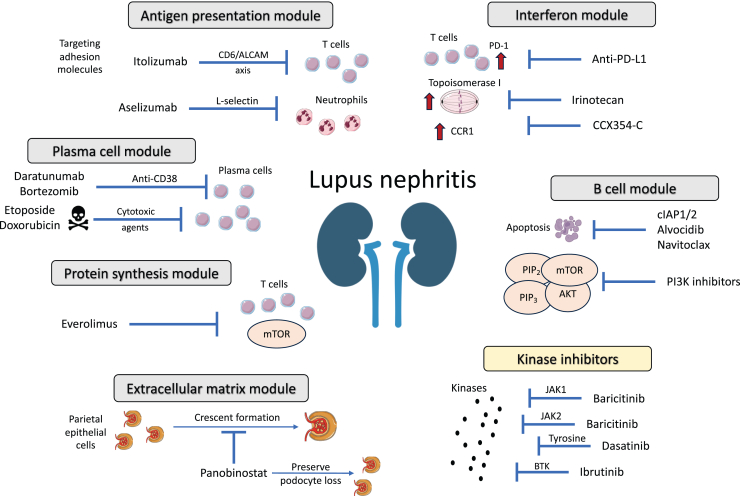
Overview of major mechanisms and effector pathways implicated in lupus nephritis pathogenesis emerging as targets of future therapies. Molecular network analysis followed by druggability assessment suggested key dysregulated gene modules involving “interferon”, “B cells”, “plasma cells”, “antigen presentation”, “protein synthesis”, and “extracellular matrix”. Aberrant transcriptomic signatures could be reversed by specific drugs, as schematically depicted. ALCAM, activated leukocyte cell adhesion molecule; Anti-PD-L1, antiprogrammed death-ligand 1; BTK, Bruton’s tyrosine kinase; CCR1, CC-motif chemokine receptor 1; cIAP1/2, cellular inhibitors of apoptosis 1/2; JAK2, Janus kinase 2; PI3K, phosphoinositide 3-kinase; TYK2, tyrosine kinase 2.

Next, we determined the anticipated response of each one of the 3 LN subgroups to therapies against targets emerging from the preceding druggability analysis. The response scores upon *in silico* modulation of selected drug targets are detailed in the Supplementary Table S34. A greater proportion of patients in the hi-IFN subgroup (73.7%) exhibited an anticipated benefit from anifrolumab (i.e., anti-IFNAR) compared with the im-IFN subgroup (11.1%; *P* = 0.004), and a numerically greater proportion compared with the lo-IFN subgroup (38.5%; *P* = 0.104). Greater proportions in the im-IFN (44.4%) and hi-IFN (68.4%) subgroups, both characterized by upregulation of the plasma cell module, exhibited an anticipated benefit to daratumumab (anti-CD38) compared with the lo-IFN subgroup (0.0%; *P* = 0.017 and *P ≤* 0.001, respectively). B-cell targeted therapies (anti-CD19) and Bruton’s tyrosine kinase inhibitors exhibited capacity to induce response to a greater extent in patients within the im-IFN subgroup, which is marked by upregulation of the B-cell module (Figure 6). Lastly, the lo-IFN subgroup exhibited a greater anticipated response to calcineurin, mammalian target of rapamycin complex 1 (mTORC1), and tyrosine kinase inhibitors compared with the other 2 subgroups (Figure 6; Supplementary Table S35). Implications from druggability analysis results are illustrated in Figure 7.

## Discussion

Tailored treatment strategies informed by immunologic aberrations are eagerly needed in LN to advance management. In this whole-blood transcriptome analysis, patients with SLE who manifested an active LN were characterized by a transcriptome signature comprising 2925 unique DEGs. Through WGCNA analysis, we identified dysregulated gene modules with implications of distinct pathogenetic mechanisms, and 3 subgroups of patients with LN representing distinct phases of the inflammatory process during active LN. We demonstrated correlations between specific gene modules and serologic markers, including proinflammatory cytokines and novel autoantibodies. Subsequently, molecular network analysis followed by druggability assessment and *in silico* predicted response suggested new potential targets of therapy as well as potentiality to repurpose existing drugs, which could be amenable to challenge in LN clinical trials.

To improve our understanding of the immune pathway heterogeneity underlying LN toward precision medicine approaches, we identified key mechanisms of importance in patients with active LN. First, we discerned an LN-specific gene signature, which corroborated an enhanced neutrophil degranulation.^30^^,^^31^ Next, we demonstrated 20 distinct dysregulated gene modules involving both innate and adaptive immune pathways, similar to previous implications in adult and pediatric SLE,^32^ which grouped the patients with LN into 3 subgroups, likely representing different phases of a renal flare in SLE. These subgroups were primarily classified based on the degree of upregulation of the IFN gene module and were termed lo-IFN, im-IFN, and hi-IFN patient subgroups. Interestingly, the lo-IFN patient subgroup displayed a prominent downregulation of the “B cell” and “plasma cells, Ig” gene modules. This subgroup exhibited anticipated benefit from calcineurin (e.g., voclosporin), mammalian target of rapamycin complex 1, and tyrosine kinase inhibitors. Conversely, the im-IFN subgroup exhibited upregulation of the “B cell” and “plasma cells, Ig” gene modules and was predicted to benefit from B-cell and plasma-cell targeting therapies such as anti-CD19 and anti-CD38, respectively. These findings are of particular importance in light of recent implications for anti-CD19 CAR T cell therapy^33^ and daratumumab^34^^,^^35^ for treating refractory LN. Lastly, the hi-IFN subgroup was characterized by downregulation of the “B cell” gene module and upregulation of the “plasma cells, Ig” module and demonstrated an anticipated benefit from type I IFN receptor inhibitors (e.g., anifrolumab). Indeed, all these pathways are considered major mediators of LN.^36^^,^^37^ The IFN signature is strongly associated with both active and inactive LN.^38^^,^^39^ IFN-related genes are overexpressed in kidney biopsies, and plasmacytoid dendritic cells (key IFN-producing cells) infiltrate glomeruli in patients with active LN.^40^^,^^41^ More importantly, IFN-β induces apoptosis to podocytes, whereas type I IFNs directly inhibit renal stem or progenitor cells from differentiation into podocytes, resulting in inadequate glomerular repair.^42^ It is important to emphasize that these patient subgroups may not necessarily represent distinct molecular LN entities but rather phases of the inflammation during a renal flare. Nonetheless, these subsets could benefit differentially from different types of therapies, pointing toward the potential of personalized medicine in LN.

IFN-α *per se* stimulates B-cell differentiation toward antibody-producing plasma cells.^43^ B cells promote autoimmunity via autoantibody production, which leads to immune complex formation and immune complex recognition by Fc-γ receptors. These cells also exert other properties, including enhanced antigen presentation to T cells, increased expression of toll-like receptors, and increased secretion of proinflammatory cytokines.^37^ These pathways have gained traction as promising therapeutic targets in LN over the past decade.^44^ A phase 2 randomized controlled trial (RCT) of the type I IFN inhibitor anifrolumab in patients with active LN did not meet its primary end point; however, the use of anifrolumab was associated with improvements in various secondary end points, including complete renal response, warranting further investigation.^45^ Encouraging real-life data on the anti-CD20 B-cell depletory monoclonal rituximab led to the first phase 3 RCT testing the efficacy of rituximab as an add-on therapy to mycophenolate in active proliferative LN (LUNAR).^46^ Although this trial did not meet its primary end point, off-label use of rituximab is recommended in refractory cases of LN based on accumulating evidence from observational studies and *post hoc* analyses of clinical trial data.^5^^,^^47^^,^^48^ A recent phase 2 RCT of a humanized anti-CD20 monoclonal antibody (obinutuzumab) encompassing 125 patients with LN showed superiority of obinutuzumab over placebo in inducing complete renal response.^49^ The monoclonal belimumab against the soluble counterpart of B-cell activating factor (BAFF)^37^ was recently approved for the treatment of active LN on top of mycophenolic acid or low-dose cyclophosphamide. Collectively, B-cell targeting therapies appear effective in LN,^37^ whereas RCTs on IFN inhibition are pending.

Following weighted gene coexpression network analysis, we identified correlations between the dysregulation of replicated gene modules and specific serologic markers. Particularly, IgM antibodies against phosphorylcholine and antimalondialdehyde were negatively associated with the “platelets” module. Antiphosphorylcholine but not antimalondialdehyde antibodies have been shown to promote T regulatory cell polarization and are negatively correlated with cardiovascular events in autoimmune diseases, suggesting that they may exert protective effects through regulation of platelets.^50^ IL-6 was positively correlated with the “monocytes” and “plasma cells, Ig” modules, whereas elevated levels of TNF-α were associated with the “interferon (II)” and “plasma cells, Ig” modules. Monocytes are considered the main source of IL-6 in SLE, whereas IL-6 enhances B-cell differentiation,^51^ and TNF-α contributes to the maintenance of long-lived plasma cell niches, which play a key role in SLE pathogenesis.^52^ Moreover, TNF-α directly regulates the production and secretion of IFN-α by plasmacytoid dendritic cells.^53^ These data further support the use of IFN targeting drugs in LN.

Scrutinizing the “interferon” module, we generated gene-based signaling molecule networks to identify chief regulators and downstream therapeutic targets. Topoisomerase 1, CCR1, and PD-L1 were among the most promising targets to inhibit. Irinotecan is a topoisomerase 1 inhibitor that has been shown to ameliorate LN by altering DNA relaxation and anti-dsDNA binding in 2 different mouse models of lupus.^54^, ^55^, ^56^ Interestingly, low-dose irinotecan was administrated as add-on therapy in a patient with refractory mixed proliferative and membranous LN with favorable outcomes and no safety concerns.^57^ CCR1 inhibition with the oral antagonist BL5923 has been tested in murine lupus demonstrating reduced T cell and macrophage infiltration in the kidneys and significant clinical improvement.^58^ The oral CCR1 inhibitor, CCX354-C, was investigated in a phase 2 RTC of rheumatoid arthritis and was tolerable and safe but failed to yield clinical benefit.^59^ PD-L1 antagonists, also termed immune checkpoint inhibitors, dramatically changed the natural history of some cancers. Side-effects of these inhibitors include immune-related manifestations, including lupus-like disease.^60^ Intriguingly, our data indicate that anti-PD-L1 might be beneficial in LN. These findings are consistent with SLE pathogenesis; toll-like receptors and type I IFN directly regulate the expression of PD-1/PD-L1 via activation of signal transducer and activator of transcription 1 and/or NF-κB.^61^ Patients with SLE display aberrant regulation of the PD-1/PD-L1 axis, and the PD1.3 polymorphism which some patients with SLE carry has been associated with reduced transcriptional activity and PD-1 expression in activated T cells. PD-1 is expressed in glomeruli and renal tubules in patients with LN, indicating a crosstalk between the kidney and peripheral T cell immune tolerance.^62^ These therapies hold particular promise to benefit the herein identified hi-IFN LN patient subgroup. Taken together, clinical trials are needed to determine the potential efficacy of irinotecan and CCR1 inhibitors, whereas the PD-1/PD-L1 axis appears crucial in LN pathogenesis and may reveal new targets of therapy.

B cell-based druggability analysis unveiled 2 noteworthy albeit non–B-cell–specific targets, namely cellular inhibitors of apoptosis 1/2 and PI3Ks. Inhibition of cellular inhibitors of apoptosis 1/2 exhibits anti-inflammatory effects through modulation of the equilibrium between Th17 and Tregs. Notably, cellular inhibitors of apoptosis 1/2 were shown to abolish the generation of IL-17A by fostering differentiation of T cells toward Tregs.^63^ PI3Ks function as enzymes in downstream signaling pathways that govern cellular processes such as survival, proliferation, and migration. Administration of PI3Kδ inhibitors in murine LN reduced the proportion of CD4+ effector/memory cells and B cells and serum levels of TNF-α, and diminished kidney infiltration by macrophages.^64^ The potential of these drugs was herein implied for the im-IFN LN subset.

The “extracellular matrix” module uncovered potential drugs involved in the modulation of gene expression by histone deacetylases, including fimepinostat, romidepsin, entinostat, and panobinostat. Panobinostat is an oral inhibitor of histone deacetylase, recently suggested as a potential option in rapidly progressive glomerulonephritis; panobinostat acts on parietal epithelial cells and promotes their differentiation into podocytes, preventing the formation of crescents and podocyte loss.^65^ Kinases such as serine-threonine kinases and JAK2 have also emerged as potential targets in this context. Enzastaurin is an oral serine-threonine kinase inhibitor that effectively inhibits the signaling of protein kinase Cβ and PI3K/AKT pathways. In lupus-prone mice, the deficiency of protein kinase Cβ has been shown to attenuate LN, being evident with the reduced levels of autoantibodies and proteinuria as well as improved histologic features. Moreover, prophylactic treatment with enzastaurin effectively prevented the development of lupus-like disease in mice.^66^ The JAK/STAT pathway has been long investigated in autoimmune diseases.^67^ Baricitinib, a JAK1/2 inhibitor, induced improvements in lupus rashes and arthritis in a phase 2 and a phase 3 RCT.^68^ However, these results were not replicated in another phase 3 clinical trial,^69^ warranting further investigation. Deucravacitinib, another JAK inhibitor that selectively inhibits TYK2, demonstrated superiority over placebo in a phase 2 trial of SLE.^70^ Larger phase 3 trials are planned to further explore the efficacy and safety of deucravacitinib in the treatment of SLE (NCT05620407, NCT05617677), and based on our data, its potential in LN may be worth exploring.

Other dysregulated pathways in patients with LN included the cell cycle, protein synthesis, and plasma cells. Aberrant cell cycle activation can be effectively reversed by the second-generation tyrosine kinase inhibitor, dasatinib. This drug is approved for the treatment of Philadelphia chromosome-positive acute lymphoblastic leukemia and chronic myeloid leukemia.^71^ Dasatinib acts as a potent inhibitor of tyrosine kinases, thereby interfering with the signaling pathways that drive abnormal cell proliferation. Selinexor, a selective inhibitor of nuclear transport compounds that herein showed potential in LN based on its ability to counteract cell cycle-related aberrancies, is currently approved for the treatment of multiple myeloma in combination with bortezomib and dexamethasone.^72^ Importantly, bortezomib stands out as a highly promising therapeutic target in LN, primarily based on aberrant gene expression of PSMB2. Administration of bortezomib in 12 patients with refractory LN who previously had been treated with cyclophosphamide, mycophenolate, and rituximab resulted in partial or complete response in 11 of those 12 patients, highlighting its effectiveness and potentiality as a treatment option in refractory LN.^73^ In addition, carfilzomib, marizomib, ixazomib citrate, and oprozomib have potential in targeting pathways affected by abnormal PSMB2 expression, and based on our findings, they may be speculated to have merit in the context of LN.

Everolimus emerged from the “protein synthesis” module as an inhibitor of mammalian target of rapamycin.^74^ This compound has been clinically applied as an immunosuppressant, effectively preventing the rejection of organ transplants.^74^ By targeting mammalian target of rapamycin, everolimus modulates key cellular processes involved in protein synthesis and cell growth, thereby exerting immunosuppressive effects.

The anti-CD38 monoclonal daratumumab appeared to have ability to counteract the aberrant transcriptome signature associated with the “plasma cells” module. Daratumumab was successfully administered to 2 patients with lupus with life-threatening disease and yielded reductions in long-lived plasma cell, type I IFN, and T cell signatures.^75^ A phase 2 RCT of daratumumab in patients with active LN is ongoing (NCT04868838). The efficacy of daratumumab may be particularly enhanced in the im-IFN and hi-IFN LN patient subgroups, which exhibited distinct upregulation of the "plasma cells, Ig" gene module. Alvocidib, a synthetic flavonoid compound deriving from an extract of an Indian plant, exhibits ability to inhibit cyclin-dependent kinases, resulting in cell division arrest and induction of apoptosis.^76^ Herein, it showed potential in reversing the plasma cell-mediated molecular signature. To this end, dysregulation patterns within the “plasma cells” module revealed merit for a plethora of cytotoxic drugs, such as daunorubicin, doxorubicin, and etoposide. These drugs could potentially be explored as rescue therapies in refractory and/or life-threatening cases.

Analysis of the “regulation of antigen presentation” gene module uncovered additional intriguing targets. Drugs with antiapoptotic properties including Bcl-2 inhibitors, (e.g., navitoclax), or irreversible inhibitors of caspases 1, 8, and 9, (e.g., nivocasan), showed promise in modulating antigen presentation processes. In addition, targeting the cell adhesion molecule (CAM) L-selectin with aselizumab has shown promise in regulating the migration of neutrophils. L-selectin is a CAM expressed in circulating neutrophils and plays a crucial role in their chemotaxis toward sites of inflammation.^77^ Considering that neutrophils are key mediators in LN, targeting L-selectin may potentially be an effective approach.^78^ Importantly, we observed dysregulated gene expression of CD6 and activated leukocyte CAM. A recent study showed that the CD6/activated leukocyte CAM pathway is a key orchestrator of LN through activation of T cell responses.^79^ More specifically, activated leukocyte CAM (ALCAM) is expressed in renal cells, whereas CD6 is expressed only by T cells. Patients with LN carry an increased proportion of CD6+/ALCAM+ cell subpopulations, indicating that this pathway plays a role in T cell trafficking into the kidneys. Blocking CD6 in murine lupus resulted in significant reductions in immune cell infiltration and amelioration of nephritis. Itolizumab, a humanized recombinant anti-CD6 monoclonal antibody, yielded favorable outcomes in psoriasis with no safety signals.^80^ Other promising targets identified within this module included Bruton’s tyrosine kinase inhibitors (e.g., ibrutinib). These inhibitors have been extensively studied in the context of autoimmune diseases due to their multifaceted mechanism of action, targeting both B cells and macrophages. Bruton’s tyrosine kinase inhibitors have been evaluated in murine lupus yielding improvements in serologic markers, glomerulonephritis, skin disease, and neuropsychiatric manifestations.^81^^,^^82^ However, phase 2 RCTs failed to demonstrate benefit from Bruton’s tyrosine kinase inhibitors in active extrarenal SLE.^83^ Nevertheless, this research avenue may hold promise in the development of novel therapeutic strategies for LN.

Our study has certain limitations that need to be acknowledged. First, because all patients were of European ancestry, our results cannot be broadly generalized to other ethnic groups. Multinational validation is anticipated through the ongoing study “Per-protocol repeat kidney biopsy in incident cases of lupus nephritis” (ReBioLup; http://rebiolup.com; NCT04449991). The cross-sectional design did not allow us to investigate intraindividual changes of the transcriptome. It is important to acknowledge that some selection bias was imposed by exclusion criteria such as nephrotic-range proteinuria, mycophenolate mofetil equivalents >2 g/d, i.v. GCs, and prednisone equivalents >15 mg/d within the preceding 3 months from the sampling occasion, which likely limits our cases of renal SLE to less severe or not full-blown renal flares. Moreover, no kidney biopsy was performed in proximity to the sampling but in 2 cases. However, our definition of current renal activity at the time of sampling was based on a history of a biopsy-proven LN and current proteinuria levels ≥0.5 g/d in all cases. In addition, proteinuria was recorded at the time of sampling and the corresponding clinical visit for the purpose of the study; thus, before potential treatment adjustments for mitigating renal activity. It is also worth noting that the GC restrictions imposed by the study protocol have the advantage of limited interference with gene expression. Analyses of cytokine and autoantibody profiles were not adjusted for multiple testing. Importantly, the druggability analysis does not differentiate between systemic and in situ effects of candidate drugs. Lastly, our findings derived from whole-blood RNA-sequencing; this limited us from identification of specific immune cell populations underlying observed transcriptome signatures, which would have helped reveal potential cell-targeted therapies. Single-cell RNA sequencing from key immune cells would provide complemental information to our findings toward a more in-depth understanding of the molecular signatures underlying LN. Overall, the results are considered hypothesis-generating and should be interpreted with caution. Among strengths of the present investigation was the careful selection of patients with SLE with active LN at the time of sampling, ensuring a homogeneous and well-characterized patient population. Our unsupervised analysis identified distinct molecular subsets, which coupled with the druggability analysis yielded cluster-specific and patient subgroup-specific implications, paving the way toward precision medicine in LN.

In conclusion, we herein report distinct and cluster-specific mechanisms underlying renal disease owing to SLE in a clinically homogenous cohort, with implications toward personalized medicine. Gene expression patterns or proxies for gene dysregulation could be used in clinical practice to stratify patients into subsets with unique anticipated responses to either existing or novel targeted therapies, paving the way for informed and tailored treatment strategies in LN. Of particular interest were irinotecan, CCR1 inhibitors, and PD-L1 antagonists interfering with IFN-mediated pathways; panobinostat, which promotes differentiation of renal progenitor cells into podocytes with properties altering the extracellular matrix signaling; and bortezomib and daratumumab for their interference with plasma cells. In addition, our data reveal novel potential targets whose modulation could reverse key aberrant pathways in LN, such as IFN and B-cell signaling. Although further studies are warranted to investigate the therapeutic potential of these drugs, our findings contribute to a conceptual framework of precision medicine in the future management of LN.

## Appendix

### List of PRECISESADS Clinical Consortium

Lorenzo Beretta, Barbara Vigone, Jacques-Olivier Pers, Alain Saraux, Valérie Devauchelle-Pensec, Divi Cornec, Sandrine Jousse-Joulin, Bernard Lauwerys, Julie Ducreux, Anne-Lise Maudoux, Carlos Vasconcelos, Ana Tavares, Esmeralda Neves, Raquel Faria, Mariana Brandão, Ana Campar, António Marinho, Fátima Farinha, Isabel Almeida, Miguel Angel Gonzalez-Gay Mantecón, Ricardo Blanco Alonso, Alfonso Corrales Martínez, Ricard Cervera, Ignasi Rodríguez-Pintó, Gerard Espinosa, Rik Lories, Ellen De Langhe, Nicolas Hunzelmann, Doreen Belz, Torsten Witte, Niklas Baerlecken, Georg Stummvoll, Michael Zauner, Michaela Lehner, Eduardo Collantes, Rafaela Ortega-Castro, Ma Angeles Aguirre-Zamorano, Alejandro Escudero-Contreras, Ma Carmen Castro -Villegas, Norberto Ortego, María Concepción Fernández Roldán, Enrique Raya, Inmaculada Jiménez Moleón, Enrique de Ramon, Isabel Díaz Quintero, Pier Luigi Meroni, Maria Gerosa, Tommaso Schioppo, Carolina Artusi, Carlo Chizzolini, Aleksandra Zuber, Donatienne Wynar, Laszló Kovács, Attila Balog, Magdolna Deák, Márta Bocskai, Sonja Dulic, Gabriella Kádár, Falk Hiepe, Velia Gerl, Silvia Thiel, Manuel Rodriguez Maresca, Antonio López-Berrio, Rocío Aguilar-Quesada, and Héctor Navarro-Linares.

## Disclosure

IP has received research funding and/or honoraria from Amgen, AstraZeneca, Aurinia, BMS, Elli Lilly, Gilead, GSK, Janssen, Novartis, Otsuka, and Roche. All the other authors declared no competing interests.

## Supplementary Material

Supplementary Material (PDF)

Supplementary File (Excel)

**List of Local Investigators From the Participating Clinical Sites.**

**Figure S1.** The NRF1 signaling molecule network and annotated drug targets in patients with lupus nephritis.

**Figure S2.** The YY1 signaling molecule network and annotated drug targets in patients with lupus nephritis.

**Figure S3.** The ZBTB33 signaling molecule network and annotated drug targets in patients with lupus nephritis.

**Figure S4.** The PRDM2 signaling molecule network and annotated drug targets in patients with lupus nephritis.

**Table S1.** Main inclusion and exclusion criteria.

**Table S2.** Modified STROBE statement checklist.

**Table S3.** Renal disease activity in lupus nephritis subgroups.

**Table S4.** Correlations between serologic markers and z-scores of the “B cell” gene module in patients with lupus nephritis.

**Table S5.** Correlations between serologic markers and *z*-scores of the “cell cycle, mitotic phase” gene module in patients with lupus nephritis.

**Table S6.** Correlations between serologic markers and *z*-scores of the “CORO1A-DEF6 network” gene module in patients with lupus nephritis.

**Table S7.** Correlations between serologic markers and *z*-scores of the “enriched in antigen presentation (III)” gene module in patients with lupus nephritis.

**Table S8.** Correlations between serologic markers and *z*-scores of the “enriched in neutrophils (II)” gene module in patients with lupus nephritis.

**Table S9.** Correlations between serologic markers and *z*-scores of the “erythrocytes” gene module in patients with lupus nephritis.

**Table S10.** Correlations between serologic markers and *z*-scores of the “extracellular matrix (I)” gene module in patients with lupus nephritis.

**Table S11.** Correlations between serologic markers and z-scores of the “extracellular region cluster” gene module in patients with lupus nephritis.

**Table S12.** Correlations between serologic markers and *z*-scores of the “inflammation (IV)” gene module in patients with lupus nephritis.

**Table S13.** Correlations between serologic markers and z-scores of the “inflammation (VI)” gene module in patients with lupus nephritis.

**Table S14.** Correlations between serologic markers and z-scores of the “inositol phosphate metabolism” gene module in patients with lupus nephritis.

**Table S15.** Correlations between serologic markers and *z*-scores of the “interferon” gene module in patients with lupus nephritis.

**Table S16.** Correlations between serologic markers and *z*-scores of the “interferon (II)” gene module in patients with lupus nephritis.

**Table S17.** Correlations between serologic markers and *z*-scores of the “MAPK, RAS signaling” gene module in patients with lupus nephritis.

**Table S18.** Correlations between serologic markers and *z*-scores of the “monocytes” gene module in patients with lupus nephritis.

**Table S19.** Correlations between serologic markers and *z*-scores of the “plasma cells, Ig” gene module in patients with lupus nephritis.

**Table S20.** Correlations between serologic markers and *z*-scores of the “platelets” gene module in patients with lupus nephritis.

**Table S21.** Correlations between serologic markers and *z*-scores of the “protein synthesis (I)” gene module in patients with lupus nephritis.

**Table S22.** Correlations between serologic markers and *z*-scores of the “regulation of antigen presentation and immune response” gene module in patients with lupus nephritis.

**Table S23.** Correlations between serologic markers and *z*-scores of the “regulation of transcription, transcription factors” gene module in patients with lupus nephritis.

**Table S24.***Z*-scores of replicated gene modules in patients with lupus nephritis and anti-βGPI IgG positivity versus negative patients.

**Table S25.***Z*-scores of replicated gene modules in patients with lupus nephritis and aCL IgG positivity versus negative patients.

**Table S26.***Z*-scores of replicated gene modules in patients with lupus nephritis and low versus normal/high levels of C3c.

**Table S27.***Z*-scores of replicated gene modules in patients with lupus nephritis and low versus normal/high levels of C4.

**Table S28.***Z*-scores of replicated gene modules in patients with lupus nephritis and antichromatin positivity versus negative patients.

**Table S29.***Z*-scores of replicated gene modules in patients with lupus nephritis and anti-dsDNA positivity versus negative patients.

**Table S30.***Z*-scores of replicated gene modules in patients with lupus nephritis and anti-SSA-52 positivity versus negative patients.

**Table S31.***Z*-scores of replicated gene modules in patients with lupus nephritis and anti-SSA-60 positivity versus negative patients.

**Table S32.***Z*-scores of replicated gene modules in patients with lupus nephritis and anti-U1RNP positivity versus negative patients.

**Table S33.** The most enriched signaling molecule networks in patients with lupus nephritis.

**Table S34.** Response scores to selected targets in patients with lupus nephritis.

**Table S35.** Patients with lupus nephritis with anticipated response to selected drug targets.

**Sheet 1.** Differentially expressed genes in patients with active lupus nephritis versus healthy controls.

**Sheet 2.** Differentially expressed genes in patients with active nonrenal systemic lupus erythematosus versus healthy controls.

**Sheet 3.** Enriched biological process gene ontology terms from overrepresentation analysis of lupus nephritis-specific differentially expressed genes in patients with active lupus nephritis versus healthy controls.

**Sheet 4.** Enriched molecular function gene ontology terms from overrepresentation analysis of lupus nephritis-specific differentially expressed genes in patients with active lupus nephritis versus healthy controls.

**Sheet 5.** Enriched Reactome pathways from overrepresentation analysis of lupus nephritis-specific differentially expressed genes in patients with active lupus nephritis versus healthy controls.

**Sheet 6.** Replicated gene modules in patients with lupus nephritis.

**Sheet 7.** Genes included in replicated gene modules in patients with lupus nephritis.

**Sheet 8.** Pearson correlations between replicated gene modules and immune modules in patients with lupus nephritis.

**Sheet 9.** Networks and drug targets in patients with lupus nephritis.
