# Supplementary material for: Interferon and B-cell Signatures Inform Precision Medicine in Lupus Nephritis
Source: Kidney Int Rep. 2024 Mar 13;9(6):1817–35. doi: 10.1016/j.ekir.2024.03.014 (PMC11184261; doi:10.1016/j.ekir.2024.03.014)
Supplement: Supplementary Material (PDF) [file mmc1.pdf]

Supplementary Material

Table of contents

Supplementary Table S1. Main inclusion and exclusion criteria..4

Supplementary Table S2. Modified STROBE statement checklist 5

List of local investigators from the participating clinical sites 8

Supplementary Table S3. Renal disease activity in LN subgroups 9

Supplementary Table S4. Correlations between serological markers and z-scores of the “B cell” gene module in patients with LN...10

Supplementary Table S5. Correlations between serological markers and z-scores of the “cell cycle, mitotic phase” gene module in patients with LN...11

Supplementary Table S6. Correlations between serological markers and z-scores of the “CORO1A-DEF6 network” gene module in patients with LN...12

Supplementary Table S7. Correlations between serological markers and z-scores of the “enriched in antigen presentation (III)” gene module in patients with LN...............................13

Supplementary Table S8. Correlations between serological markers and z-scores of the “enriched in neutrophils (II)” gene module in patients with LN 14

Supplementary Table S9. Correlations between serological markers and z-scores of the “erythrocytes” gene module in patients with LN..15

Supplementary Table S10. Correlations between serological markers and z-scores of the “extracellular matrix (I)” gene module in patients with LN 16

Supplementary Table S11. Correlations between serological markers and z-scores of the “extracellular region cluster” gene module in patients with LN...17

Supplementary Table S12. Correlations between serological markers and z-scores of the “inflammation (IV)” gene module in patients with LN 18

Supplementary Table S13. Correlations between serological markers and z-scores of the “inflammation (VI)” gene module in patients with LN 19

Supplementary Table S14. Correlations between serological markers and z-scores of the “inositol phosphate metabolism” gene module in patients with LN...20

Supplementary Table S15. Correlations between serological markers and z-scores of the “interferon” gene module in patients with LN..21

Supplementary Table S16. Correlations between serological markers and z-scores of the “interferon (II)” gene module in patients with LN..22

Supplementary Table S17. Correlations between serological markers and z-scores of the “MAPK, RAS signalling” gene module in patients with LN..23

Supplementary Table S18. Correlations between serological markers and z-scores of the “monocytes” gene module in patients with LN 24
Supplementary Table S19. Correlations between serological markers and z-scores of the “plasma cells, immunoglobulins” gene module in patients with LN....................25
Supplementary Table S20. Correlations between serological markers and z-scores of the “platelets” gene module in patients with LN 26
Supplementary Table S21. Correlations between serological markers and z-scores of the “protein synthesis (I)” gene module in patients with LN....................27
Supplementary Table S22. Correlations between serological markers and z-scores of the “regulation of antigen presentation and immune response” gene module in patients with LN.28
Supplementary Table S23. Correlations between serological markers and z-scores of the “regulation of transcription, transcription factors” gene module in patients with LN..............29
Supplementary Table S24. Z-scores of replicated gene modules in patients with LN and anti-βGPI IgG positivity versus negative patients....................30
Supplementary Table S25. Z-scores of replicated gene modules in patients with LN and aCL IgG positivity versus negative patients 31
Supplementary Table S26. Z-scores of replicated gene modules in patients with LN and low versus normal/high levels of C3c....................32
Supplementary Table S27. Z-scores of replicated gene modules in patients with LN and low versus normal/high levels of C4....................33
Supplementary Table S28. Z-scores of replicated gene modules in patients with LN and anti-chromatin positivity versus negative patients 34
Supplementary Table S29. Z-scores of replicated gene modules in patients with LN and anti-dsDNA positivity versus negative patients 35
Supplementary Table S30. Z-scores of replicated gene modules in patients with LN and anti-SSA-52 positivity versus negative patients....................36
Supplementary Table S31. Z-scores of replicated gene modules in patients with LN and anti-SSA-60 positivity versus negative patients....................37
Supplementary Table S32. Z-scores of replicated gene modules in patients with LN and anti-U1RNP positivity versus negative patients....................38
Supplementary Table S33. The most enriched signalling molecule networks in patients with LN 39
Supplementary Figure S1. The *NRF1* signalling molecule network and annotated drug targets in patients with lupus nephritis 40
Supplementary Figure S2. The *YY1* signalling molecule network and annotated drug targets in patients with lupus nephritis....................41
Supplementary Figure S3. The *ZBTB33* signalling molecule network and annotated drug targets in patients with lupus nephritis....................42
Supplementary Figure S4. The *PRDM2* signalling molecule network and annotated drug targets in patients with lupus nephritis....................43

Supplementary Table S34. Response scores to selected targets in patients with LN 44
Supplementary Table S35. LN patients with anticipated response to selected drug targets......45

Supplementary Table S1. Main inclusion and exclusion criteria

Inclusion criteria	Exclusion criteria
**For all patients**	**For all patients**
- Age ≥18 years<br>- Diagnosed according to the prevailing criteria for systemic lupus erythematosus (SLE)<br>- Informed consent signed	- Pediatric lupus<br>- Drug-induced lupus<br>- Severe nephrotic syndrome with proteinuria ≥3.5 g/day<br>- Patients with stable doses of prednisone equivalent >15 mg/day for the last 3 months or with IV corticosteroids in the last 3 months<br>- Patients under immunosuppressant treatment in the last 3 months prior to recruitment and patients with combined therapy using two or more immunosuppressants:<br>□ Methotrexate ≥25mg/week<br>□ Azathioprine ≥2.5mg/kg/day<br>□ Cyclosporine A >3mg/kg/day<br>□ Mycophenolate mofetil >2g/day<br>- Chronic HBV or HCV infection<br>- Patients who are also diagnosed according to the prevailing criteria for one of the following autoimmune diseases:<br>□ Rheumatoid arthritis (RA)<br>□ Scleroderma or systemic sclerosis (SSc)<br>□ Primary Sjögren's syndrome (pSjS)<br>□ Primary antiphospholipid syndrome (pAPS)<br>□ Mixed connective tissue disease (MCTD)<br>□ Patients with undifferentiated connective tissue disease (UCTD) for over 1 year and that do not fulfill the diagnosis of any of the above diseases or SLE
	**For controls**
	- Individuals on chronic medication<br>- Individuals suffering from any inflammatory autoimmune, allergic or infectious condition, and with a history of autoimmune disease, particularly thyroid disease or other diseases that may modify cellular profiles in blood

Adapted from "Integrative Analysis Reveals a Molecular Stratification of Systemic Autoimmune Diseases." Arthritis Rheum. 2021;73(6):1073-1085. Copyright 2021 by the American College of Rheumatology. Adapted with permission. IV: intravenous, HBV: hepatitis B virus, HCV: hepatitis C virus.

Supplementary Table S2. Modified STROBE statement checklist

Section(s)	Item No	Recommendation	Page No
Title and abstract	1	(*a*) Indicate the study's design with a commonly used term in the title or the abstract	
		(*b*) Provide in the abstract an informative and balanced summary of what was done and what was found	3
Introduction			
Background/rationale	2	Explain the scientific background and rationale for the investigation being reported	4–5
Objectives	3	State specific objectives, including any prespecified hypotheses	5
Methods			
Study design	4	Present key elements of study design early in the paper	5–8
Setting	5	Describe the setting, locations, and relevant dates, including periods of recruitment, exposure, follow-up, and data collection	5–6
Participants	6	(*a*) *Cohort study*—Give the eligibility criteria, and the sources and methods of selection of participants. Describe methods of follow-up<br>*Case-control study*—Give the eligibility criteria, and the sources and methods of case ascertainment and control selection. Give the rationale for the choice of cases and controls<br>*Cross-sectional study*—Give the eligibility criteria, and the sources and methods of selection of participants	5; Supplementary Material
Variables	7	Clearly define all outcomes, exposures, predictors, potential confounders, and effect modifiers. Give diagnostic criteria, if applicable	5–8
Data sources/ measurement	8*	For each variable of interest, give sources of data and details of methods of assessment (measurement).	5–8
Bias	9	Describe any efforts to address potential sources of bias	6–8
Study size	10	Explain how the study size was arrived at (if applicable)	
Quantitative variables	11	Explain how quantitative variables were handled in the analyses. If applicable, describe which groupings were chosen and why	6–8
Statistical methods	12	(*a*) Describe all statistical methods, including those used to control for confounding	6–8
		(*b*) Describe any methods used to examine subgroups and interactions	7–8

		(*c*) Explain how missing data were addressed	
		(*d*) *Cohort study*—If applicable, explain how loss to follow-up was addressed *Case-control study*—If applicable, explain how matching of cases and controls was addressed *Cross-sectional study*—If applicable, describe analytical methods taking account of sampling strategy	6–8
		(*e*) Describe any sensitivity analyses	
Results			
Participants	13*	(a) Report numbers of individuals at each stage of study—eg numbers potentially eligible, examined for eligibility, confirmed eligible, included in the study, completing follow-up, and analyzed	9–10; Supplementary Material
		(c) Use of a flow diagram	
Descriptive data	14*	(a) Give characteristics of study participants (eg demographic, clinical, social) and information on exposures and potential confounders	9
		(b) Indicate number of participants with missing data for each variable of interest	9; Supplementary Material
		(c) *Cohort study*—Summarise follow-up time (eg, average and total amount)	
Outcome data	15*	*Cohort study*—Report numbers of outcome events or summary measures over time	
		Case-control study—Report numbers in each exposure category, or summary measures of exposure	
		Cross-sectional study—Report numbers of outcome events or summary measures	9-13; Supplementary Material
Main results	16	(*a*) Give unadjusted estimates and, if applicable, confounder-adjusted estimates and their precision (eg, 95% confidence interval). Make clear which confounders were adjusted for and why they were included	9-13; Supplementary Material
Other analyses	17	Report other analyses done—eg analyses of subgroups and interactions, and sensitivity analyses	9-13; Supplementary Material
Discussion			
Key results	18	Summarise key results with reference to study objectives	13–22
Limitations	19	Discuss limitations of the study, taking into account sources of potential bias or imprecision. Discuss both direction and magnitude of any potential bias	21–22
Interpretation	20	Give a cautious overall interpretation of results considering objectives, limitations, multiplicity of analyses, results from similar studies, and other relevant evidence	13–22

Generalisability	21	Discuss the generalisability (external validity) of the study results	21–22

List of local investigators from the participating clinical sites

Lorenzo Beretta[1], Barbara Vigone[1], Jacques-Olivier Pers[2], Alain Saraux[2], Valérie Devauchelle-Pensec[2], Divi Cornec[2], Sandrine Jousse-Joulin[2], Bernard Lauwerys[3], Julie Ducreux[3], Anne-Lise Maudoux[3], Carlos Vasconcelos[4], Ana Tavares[4], Esmeralda Neves[4], Raquel Faria[4], Mariana Brandão[4], Ana Campar[4], António Marinho[4], Fátima Farinha[4], Isabel Almeida[4], Miguel Angel Gonzalez-Gay Mantecón[5], Ricardo Blanco Alonso[5], Alfonso Corrales Martínez[5], Ricard Cervera[6], Ignasi Rodríguez-Pintó[6], Gerard Espinosa[6], Rik Lories[7], Ellen De Langhe[7], Nicolas Hunzelmann[8], Doreen Belz[8], Torsten Witte[9], Niklas Baerlecken[9], Georg Stummvoll[10], Michael Zauner[10], Michaela Lehner[10], Eduardo Collantes[11], Rafaela Ortega-Castro[11], Mª Angeles Aguirre-Zamorano[11], Alejandro Escudero-Contreras[11], Mª Carmen Castro-Villegas[11], Norberto Ortego[12], María Concepción Fernández Roldán[12], Enrique Raya[13], Inmaculada Jiménez Moleón[13], Enrique de Ramon[14], Isabel Díaz Quintero[14], Pier Luigi Meroni[15], Maria Gerosa[15], Tommaso Schioppo[15], Carolina Artusi[15], Carlo Chizzolini[16], Aleksandra Zuber[16], Donatienne Wynar[16], Laszló Kovács[17], Attila Balog[17], Magdolna Deák[17], Márta Bocskai[17], Sonja Dulic[17], Gabriella Kádár[17], Falk Hiepe[18], Velia Gerl[18], Silvia Thiel[18], Manuel Rodriguez Maresca[19], Antonio López-Berrio[19], Rocío Aguilar-Quesada[19], Héctor Navarro-Linares[19]

Affiliations

1. Referral Center for Systemic Autoimmune Diseases, Fondazione IRCCS Ca' Granda Ospedale Maggiore Policlinico di Milano, Italy
2. Centre Hospitalier Universitaire de Brest, Hospital de la Cavale Blanche, Brest, France
3. Pôle de pathologies rhumatismales systémiques et inflammatoires, Institut de Recherche Expérimentale et Clinique, Université catholique de Louvain, Brussels, Belgium
4. Centro Hospitalar do Porto, Portugal
5. Servicio Cantabro de Salud, Hospital Universitario Marqués de Valdecilla, Santander, Spain
6. Hospital Clinic I Provicia, Institut d'Investigacions Biomèdiques August Pi i Sunyer, Barcelona, Spain
7. Katholieke Universiteit Leuven, Belgium
8. Klinikum der Universitaet zu Koeln, Cologne, Germany
9. Medizinische Hochschule Hannover, Germany
10. Medical University Vienna, Vienna, Austria
11. Servicio Andaluz de Salud, Hospital Universitario Reina Sofía Córdoba, Spain
12. Servicio Andaluz de Salud, Complejo hospitalario Universitario de Granada (Hospital Universitario San Cecilio), Spain
13. Servicio Andaluz de Salud, Complejo hospitalario Universitario de Granada (Hospital Virgen de las Nieves), Spain
14. Servicio Andaluz de Salud, Hospital Regional Universitario de Málaga, Spain
15. Università degli studi di Milano, Milan, Italy
16. Hospitaux Universitaires de Genève, Switzerland
17. University of Szeged, Szeged, Hungary
18. Charite, Berlin, Germany
19. Andalusian Public Health System Biobank, Granada, Spain

Supplementary Table S3. Renal disease activity in LN subgroups

**Target**	**lo-IFN subgroup N=13**	**im-IFN subgroup N=9**	**hi-IFN subgroup N=19**	**lo-IFN vs im-IFN *p* value**	**lo-IFN vs hi-IFN *p* value**	**im-IFN vs hi-IFN *p* value**
**rSLEDAI-2K**	7.38□3.59	6.67□2.00	6.95□3.49	0.822	0.692	0.871

Data are presented as mean (standard deviation). All *p* values are derived from non-parametrical Mann-Whitney *U* tests. Statistically significant *p* values are in bold.

hi: high; IFN: interferon; im: intermediate; LN: lupus nephritis; lo: low; rSLEDAI-2K: renal SLE Disease Activity Index 2000.

Supplementary Table S4. Correlations between serological markers and z-scores of the “B cell” gene module in patients with LN

Serological marker	n	Coefficient	*p* value
**anti-MDA IgM**	31	0.09	0.612
**anti-PC IgM**	31	-0.07	0.717
**BAFF**	28	0.07	0.707
**IL-6**	28	0.13	0.523
**TGF-ß**	28	-0.17	0.376
**TNF-α**	28	0.21	0.282

Spearman's rank correlation coefficients of correlations between levels of different serological markers and Z-scores of the B cell gene module. The total number of patients with available data is indicated. Statistically significant *p* values are in bold. Only comparisons with sufficient numbers of observations (n≥10) are included.

BAFF: B cell activating factor belonging to the tumour necrosis factor family; Ig: immunoglobulin; IL-6: interleukin 6; LN: lupus nephritis; MDA: malondialdehyde; PC: phosphorylcholine; TGF-β: transforming growth factor β; TNF-α: transforming growth factor α.

Supplementary Table S5. Correlations between serological markers and z-scores of the "cell cycle, mitotic phase" gene module in patients with LN

Serological marker	n	Coefficient	*p* value
**anti-MDA IgM**	31	0.23	0.206
**anti-PC IgM**	31	0.04	0.850
**BAFF**	28	-0.13	0.525
**IL-6**	28	-0.02	0.930
**TGF-ß**	28	-0.12	0.534
**TNF-α**	28	-0.11	0.572

Spearman's rank correlation coefficients of correlations between levels of different serological markers and Z-scores of the cell cycle, mitotic phase gene module. The total number of patients with available data is indicated. Statistically significant *p* values are in bold. Only comparisons with sufficient numbers of observations (n≥10) are included.

BAFF: B cell activating factor belonging to the tumour necrosis factor family; Ig: immunoglobulin; IL-6: interleukin 6; LN: lupus nephritis; MDA: malondialdehyde; PC: phosphorylcholine; TGF-β: transforming growth factor β; TNF-α: transforming growth factor α.

Supplementary Table S6. Correlations between serological markers and z-scores of the "CORO1A-DEF6 network" gene module in patients with LN

Serological marker	n	Coefficient	*p* value
**anti-MDA IgM**	31	0.33	0.067
**anti-PC IgM**	31	-0.09	0.644
**BAFF**	28	0.16	0.407
**IL-6**	28	0.22	0.262
**TGF-ß**	28	-0.23	0.242
**TNF-α**	28	0.03	0.890

Spearman's rank correlation coefficients of correlations between levels of different serological markers and Z-scores of the CORO1A-DEF6 network gene module. The total number of patients with available data is indicated. Statistically significant *p* values are in bold. Only comparisons with sufficient numbers of observations (n≥10) are included.

BAFF: B cell activating factor belonging to the tumour necrosis factor family; CORO1A-DEF6: coronin 1A-differentially expressed in FDCP 6 homolog; Ig: immunoglobulin; IL-6: interleukin 6; LN: lupus nephritis; MDA: malondialdehyde; PC: phosphorylcholine; TGF-β: transforming growth factor β; TNF-α: transforming growth factor α.

Supplementary Table S7. Correlations between serological markers and z-scores of the "enriched in antigen presentation (III)" gene module in patients with LN

Serological marker	n	Coefficient	*p* value
**anti-MDA IgM**	31	0.10	0.575
**anti-PC IgM**	31	-0.13	0.477
**BAFF**	28	0.23	0.242
**IL-6**	28	0.26	0.177
**TGF-ß**	28	-0.02	0.910
**TNF-α**	28	0.30	0.115

Spearman's rank correlation coefficients of correlations between levels of different serological markers and Z-scores of the enriched in antigen presentation (III) gene module. The total number of patients with available data is indicated. Statistically significant *p* values are in bold. Only comparisons with sufficient numbers of observations (n≥10) are included.

BAFF: B cell activating factor belonging to the tumour necrosis factor family; Ig: immunoglobulin; IL-6: interleukin 6; LN: lupus nephritis; MDA: malondialdehyde; PC: phosphorylcholine; TGF-β: transforming growth factor β; TNF-α: transforming growth factor α.

Supplementary Table S8. Correlations between serological markers and z-scores of the "enriched in neutrophils (II)" gene module in patients with LN

Serological marker	n	Coefficient	*p* value
**anti-MDA IgM**	31	-0.10	0.588
**anti-PC IgM**	31	-0.30	0.101
**BAFF**	28	0.29	0.139
**IL-6**	28	0.08	0.674
**TGF-ß**	28	0.18	0.349
**TNF-α**	28	0.00	0.996

Spearman's rank correlation coefficients of correlations between levels of different serological markers and Z-scores of the enriched in neutrophils (II) gene module. The total number of patients with available data is indicated. Statistically significant *p* values are in bold. Only comparisons with sufficient numbers of observations (n≥10) are included.

BAFF: B cell activating factor belonging to the tumour necrosis factor family; Ig: immunoglobulin; IL-6: interleukin 6; LN: lupus nephritis; MDA: malondialdehyde; PC: phosphorylcholine; TGF-β: transforming growth factor β; TNF-α: transforming growth factor α.

Supplementary Table S9. Correlations between serological markers and z-scores of the “erythrocytes” gene module in patients with LN

Serological marker	n	Coefficient	*p* value
**anti-MDA IgM**	31	-0.32	0.082
**anti-PC IgM**	31	-0.22	0.244
**BAFF**	28	0.25	0.192
**IL-6**	28	-0.06	0.750
**TGF-ß**	28	-0.11	0.587
**TNF-α**	28	-0.10	0.600

Spearman's rank correlation coefficients of correlations between levels of different serological markers and Z-scores of the erythrocytes gene module. The total number of patients with available data is indicated. Statistically significant *p* values are in bold. Only comparisons with sufficient numbers of observations (n≥10) are included.

BAFF: B cell activating factor belonging to the tumour necrosis factor family; Ig: immunoglobulin; IL-6: interleukin 6; LN: lupus nephritis; MDA: malondialdehyde; PC: phosphorylcholine; TGF-β: transforming growth factor β; TNF-α: transforming growth factor α.

Supplementary Table S10. Correlations between serological markers and z-scores of the "extracellular matrix (I)" gene module in patients with LN

Serological marker	n	Coefficient	*p* value
**anti-MDA IgM**	31	0.20	0.274
**anti-PC IgM**	31	0.02	0.921
**BAFF**	28	0.01	0.974
**IL-6**	28	-0.01	0.965
**TGF-ß**	28	-0.16	0.423
**TNF-α**	28	0.00	0.982

Spearman's rank correlation coefficients of correlations between levels of different serological markers and Z-scores of the extracellular matrix (I) gene module. The total number of patients with available data is indicated. Statistically significant *p* values are in bold. Only comparisons with sufficient numbers of observations (n≥10) are included.

BAFF: B cell activating factor belonging to the tumour necrosis factor family; Ig: immunoglobulin; IL-6: interleukin 6; LN: lupus nephritis; MDA: malondialdehyde; PC: phosphorylcholine; TGF-β: transforming growth factor β; TNF-α: transforming growth factor α.

Supplementary Table S11. Correlations between serological markers and z-scores of the "extracellular region cluster" gene module in patients with LN

Serological marker	n	Coefficient	*p* value
**anti-MDA IgM**	31	0.03	0.858
**anti-PC IgM**	31	-0.13	0.477
**BAFF**	28	0.16	0.404
**IL-6**	28	0.14	0.479
**TGF-ß**	28	0.03	0.894
**TNF-α**	28	0.15	0.453

Spearman's rank correlation coefficients of correlations between levels of different serological markers and Z-scores of the extracellular region cluster gene module. The total number of patients with available data is indicated. Statistically significant *p* values are in bold. Only comparisons with sufficient numbers of observations (n≥10) are included.

BAFF: B cell activating factor belonging to the tumour necrosis factor family; Ig: immunoglobulin; IL-6: interleukin 6; LN: lupus nephritis; MDA: malondialdehyde; PC: phosphorylcholine; TGF-β: transforming growth factor β; TNF-α: transforming growth factor α.

Supplementary Table S12. Correlations between serological markers and z-scores of the "inflammation (IV)" gene module in patients with LN

Serological marker	n	Coefficient	*p* value
**anti-MDA IgM**	31	-0.11	0.558
**anti-PC IgM**	31	0.10	0.595
**BAFF**	28	-0.25	0.201
**IL-6**	28	-0.11	0.587
**TGF-ß**	28	0.00	0.998
**TNF-α**	28	-0.21	0.273

Spearman's rank correlation coefficients of correlations between levels of different serological markers and Z-scores of the inflammation (IV) gene module. The total number of patients with available data is indicated. Statistically significant *p* values are in bold. Only comparisons with sufficient numbers of observations (n≥10) are included.

BAFF: B cell activating factor belonging to the tumour necrosis factor family; Ig: immunoglobulin; IL-6: interleukin 6; LN: lupus nephritis; MDA: malondialdehyde; PC: phosphorylcholine; TGF-β: transforming growth factor β; TNF-α: transforming growth factor α.

Supplementary Table S13. Correlations between serological markers and z-scores of the "inflammation (VI)" gene module in patients with LN

Serological marker	n	Coefficient	*p* value
**anti-MDA IgM**	31	0.03	0.867
**anti-PC IgM**	31	-0.22	0.230
**BAFF**	28	0.26	0.184
**IL-6**	28	0.26	0.173
**TGF-ß**	28	0.02	0.927
**TNF-α**	28	0.20	0.304

Spearman's rank correlation coefficients of correlations between levels of different serological markers and Z-scores of the inflammation (VI) gene module. The total number of patients with available data is indicated. Statistically significant *p* values are in bold. Only comparisons with sufficient numbers of observations ($n \geq 10$) are included.

BAFF: B cell activating factor belonging to the tumour necrosis factor family; Ig: immunoglobulin; IL-6: interleukin 6; LN: lupus nephritis; MDA: malondialdehyde; PC: phosphorylcholine; TGF-β: transforming growth factor β; TNF-α: transforming growth factor α.

Supplementary Table S14. Correlations between serological markers and z-scores of the "inositol phosphate metabolism" gene module in patients with LN

Serological marker	n	Coefficient	*p* value
**anti-MDA IgM**	31	0.08	0.685
**anti-PC IgM**	31	0.07	0.692
**BAFF**	28	-0.18	0.359
**IL-6**	28	-0.05	0.816
**TGF-ß**	28	0.04	0.853
**TNF-α**	28	-0.21	0.292

Spearman's rank correlation coefficients of correlations between levels of different serological markers and Z-scores of the inositol phosphate metabolism gene module. The total number of patients with available data is indicated. Statistically significant *p* values are in bold. Only comparisons with sufficient numbers of observations (n≥10) are included.

BAFF: B cell activating factor belonging to the tumour necrosis factor family; Ig: immunoglobulin; IL-6: interleukin 6; LN: lupus nephritis; MDA: malondialdehyde; PC: phosphorylcholine; TGF-β: transforming growth factor β; TNF-α: transforming growth factor α.

Supplementary Table S15. Correlations between serological markers and z-scores of the "interferon" gene module in patients with LN

Serological marker	n	Coefficient	*p* value
**anti-MDA IgM**	31	0.12	0.513
**anti-PC IgM**	31	-0.06	0.742
**BAFF**	28	0.16	0.426
**IL-6**	28	0.26	0.175
**TGF-ß**	28	-0.06	0.771
**TNF-α**	28	0.32	0.100

Spearman's rank correlation coefficients of correlations between levels of different serological markers and Z-scores of the interferon gene module. The total number of patients with available data is indicated. Statistically significant *p* values are in bold. Only comparisons with sufficient numbers of observations (n≥10) are included.

BAFF: B cell activating factor belonging to the tumour necrosis factor family; Ig: immunoglobulin; IL-6: interleukin 6; LN: lupus nephritis; MDA: malondialdehyde; PC: phosphorylcholine; TGF-β: transforming growth factor β; TNF-α: transforming growth factor α.

Supplementary Table S16. Correlations between serological markers and z-scores of the "interferon (II)" gene module in patients with LN

Serological marker	n	Coefficient	*p* value
**anti-MDA IgM**	31	0.06	0.768
**anti-PC IgM**	31	-0.20	0.287
**BAFF**	28	0.26	0.180
**IL-6**	28	0.36	0.063
**TGF-ß**	28	0.00	0.993
**TNF-α**	28	0.41	0.033

Spearman's rank correlation coefficients of correlations between levels of different serological markers and Z-scores of the interferon (II) gene module. The total number of patients with available data is indicated. Statistically significant *p* values are in bold. Only comparisons with sufficient numbers of observations (n≥10) are included.

BAFF: B cell activating factor belonging to the tumour necrosis factor family; Ig: immunoglobulin; IL-6: interleukin 6; LN: lupus nephritis; MDA: malondialdehyde; PC: phosphorylcholine; TGF-β: transforming growth factor β; TNF-α: transforming growth factor α.

Supplementary Table S17. Correlations between serological markers and z-scores of the "MAPK, RAS signalling" gene module in patients with LN

Serological marker	n	Coefficient	*p* value
**anti-MDA IgM**	31	-0.14	0.440
**anti-PC IgM**	31	-0.16	0.388
**BAFF**	28	0.25	0.191
**IL-6**	28	0.00	0.987
**TGF-ß**	28	0.20	0.301
**TNF-α**	28	0.02	0.910

Spearman's rank correlation coefficients of correlations between levels of different serological markers and Z-scores of the MAPK, RAS signalling gene module. The total number of patients with available data is indicated. Statistically significant *p* values are in bold. Only comparisons with sufficient numbers of observations (n≥10) are included.

BAFF: B cell activating factor belonging to the tumour necrosis factor family; Ig: immunoglobulin; IL-6: interleukin 6; LN: lupus nephritis; MAPK: mitogen-activated protein kinase; MDA: malondialdehyde; PC: phosphorylcholine; TGF-β: transforming growth factor β; TNF-α: transforming growth factor α.

Supplementary Table S18. Correlations between serological markers and z-scores of the "monocytes" gene module in patients with LN

Serological marker	n	Coefficient	*p* value
**anti-MDA IgM**	31	0.07	0.712
**anti-PC IgM**	31	-0.12	0.532
**BAFF**	28	0.14	0.480
**IL-6**	28	0.42	**0.028**
**TGF-ß**	28	0.07	0.715
**TNF-α**	28	0.38	0.047

Spearman's rank correlation coefficients of correlations between levels of different serological markers and Z-scores of the monocytes gene module. The total number of patients with available data is indicated. Statistically significant *p* values are in bold. Only comparisons with sufficient numbers of observations (n≥10) are included.

BAFF: B cell activating factor belonging to the tumour necrosis factor family; Ig: immunoglobulin; IL-6: interleukin 6; LN: lupus nephritis; MDA: malondialdehyde; PC: phosphorylcholine; TGF-β: transforming growth factor β; TNF-α: transforming growth factor α.

Supplementary Table S19. Correlations between serological markers and z-scores of the "plasma cells, immunoglobulins" gene module in patients with LN

Serological marker	n	Coefficient	*p* value
**anti-MDA IgM**	31	0.14	0.461
**anti-PC IgM**	31	-0.09	0.635
**BAFF**	28	0.14	0.470
**IL-6**	28	0.39	0.039
**TGF-ß**	28	-0.06	0.765
**TNF-α**	28	0.39	0.041

Spearman's rank correlation coefficients of correlations between levels of different serological markers and Z-scores of the plasma cells, immunoglobulins gene module. The total number of patients with available data is indicated. Statistically significant *p* values are in bold. Only comparisons with sufficient numbers of observations (n≥10) are included.

BAFF: B cell activating factor belonging to the tumour necrosis factor family; Ig: immunoglobulin; IL-6: interleukin 6; LN: lupus nephritis; MDA: malondialdehyde; PC: phosphorylcholine; TGF-β: transforming growth factor β; TNF-α: transforming growth factor α.

Supplementary Table S20. Correlations between serological markers and z-scores of the "platelets" gene module in patients with LN

Serological marker	n	Coefficient	*p* value
**anti-MDA IgM**	31	-0.51	**0.003**
**anti-PC IgM**	31	-0.42	**0.019**
**BAFF**	28	0.29	0.128
**IL-6**	28	-0.02	0.916
**TGF-ß**	28	0.14	0.491
**TNF-α**	28	-0.05	0.791

Spearman's rank correlation coefficients of correlations between levels of different serological markers and Z-scores of the platelets gene module. The total number of patients with available data is indicated. Statistically significant *p* values are in bold. Only comparisons with sufficient numbers of observations (n≥10) are included.

BAFF: B cell activating factor belonging to the tumour necrosis factor family; Ig: immunoglobulin; IL-6: interleukin 6; LN: lupus nephritis; MDA: malondialdehyde; PC: phosphorylcholine; TGF-β: transforming growth factor β; TNF-α: transforming growth factor α.

Supplementary Table S21. Correlations between serological markers and z-scores of the "protein synthesis (I)" gene module in patients with LN

Serological marker	n	Coefficient	*p* value
**anti-MDA IgM**	31	0.16	0.380
**anti-PC IgM**	31	0.03	0.865
**BAFF**	28	0.08	0.692
**IL-6**	28	0.23	0.235
**TGF-ß**	28	-0.12	0.553
**TNF-α**	28	0.18	0.370

Spearman's rank correlation coefficients of correlations between levels of different serological markers and Z-scores of the protein synthesis (I) gene module. The total number of patients with available data is indicated. Statistically significant *p* values are in bold. Only comparisons with sufficient numbers of observations (n≥10) are included.

BAFF: B cell activating factor belonging to the tumour necrosis factor family; Ig: immunoglobulin; IL-6: interleukin 6; LN: lupus nephritis; MDA: malondialdehyde; PC: phosphorylcholine; TGF-β: transforming growth factor β; TNF-α: transforming growth factor α.

Supplementary Table S22. Correlations between serological markers and z-scores of the "regulation of antigen presentation and immune response" gene module in patients with LN

Serological marker	n	Coefficient	*p* value
**anti-MDA IgM**	31	0.18	0.337
**anti-PC IgM**	31	-0.01	0.973
**BAFF**	28	0.05	0.816
**IL-6**	28	0.13	0.500
**TGF-ß**	28	-0.18	0.362
**TNF-α**	28	0.14	0.484

Spearman's rank correlation coefficients of correlations between levels of different serological markers and Z-scores of the regulation of antigen presentation and immune response gene module. The total number of patients with available data is indicated. Statistically significant *p* values are in bold. Only comparisons with sufficient numbers of observations (n≥10) are included.

BAFF: B cell activating factor belonging to the tumour necrosis factor family; Ig: immunoglobulin; IL-6: interleukin 6; LN: lupus nephritis; MDA: malondialdehyde; PC: phosphorylcholine; TGF-β: transforming growth factor β; TNF-α: transforming growth factor α.

Supplementary Table S23. Correlations between serological markers and z-scores of the "regulation of transcription, transcription factors" gene module in patients with LN

Serological marker	n	Coefficient	*p* value
**anti-MDA IgM**	31	0.21	0.249
**anti-PC IgM**	31	0.17	0.355
**BAFF**	28	-0.16	0.413
**IL-6**	28	-0.17	0.377
**TGF-ß**	28	-0.13	0.503
**TNF-α**	28	-0.29	0.130

Spearman's rank correlation coefficients of correlations between levels of different serological markers and Z-scores of the regulation of transcription, transcription factors gene module. The total number of patients with available data is indicated. Statistically significant *p* values are in bold. Only comparisons with sufficient numbers of observations (n≥10) are included.

BAFF: B cell activating factor belonging to the tumour necrosis factor family; Ig: immunoglobulin; IL-6: interleukin 6; LN: lupus nephritis; MDA: malondialdehyde; PC: phosphorylcholine; TGF-β: transforming growth factor β; TNF-α: transforming growth factor α.

Supplementary Table S24. Z-scores of replicated gene modules in patients with LN and anti-βGPI IgG positivity versus negative patients

Gene module	anti-βGPI IgG (+) N=5	anti-βGPI IgG (-) N=26	*p* value
**B cell**	-1.54 (-2.61−-1.48)	-1.47 (-2.30−-0.32)	0.707
**Cell cycle, mitotic phase**	-0.23 (-0.86−-0.06)	-0.41 (-0.86−-0.12)	0.788
**CORO1A-DEF6 network**	-0.35 (-0.37−-0.06)	-0.07 (-0.18−0.02)	0.119
**Enriched in antigen presentation (III)**	-0.37 (-0.59−-0.17)	-0.39 (-0.53−0.03)	0.452
**Enriched in neutrophils (II)**	0.17 (-0.14−0.21)	0.06 (-0.08−0.18)	0.872
**Erythrocytes**	-0.23 (-0.30−0.19)	0.23 (-0.44−0.67)	0.707
**Extracellular matrix (I)**	-1.42 (-1.43−-1.15)	-0.74 (-1.12−-0.35)	0.060
**Extracellular region cluster**	-0.28 (-0.42−0.01)	-0.08 (-0.23−0.03)	0.259
**Inflammation (IV)**	0.25 (-0.11−0.49)	0.24 (-0.01−0.44)	0.872
**Inflammation (VI)**	0.08 (-0.50−0.34)	0.01 (-0.25−0.20)	1.000
**Inositol phosphate metabolism**	-0.13 (-0.55−0.68)	-0.05 (-0.38−0.26)	0.830
**Interferon**	0.76 (0.03−1.71)	1.41 (0.05−2.02)	0.830
**Interferon (II)**	0.23 (-0.27−0.93)	0.53 (0.24−0.70)	0.591
**MAPK−RAS signalling**	0.29 (-0.65−0.35)	-0.01 (-0.43−0.33)	0.957
**Monocytes**	0.38 (0.26−0.86)	0.26 (-0.15−0.92)	0.390
**Plasma cells, immunoglobulins**	0.04 (-0.24−1.06)	-0.16 (-0.70−0.56)	0.452
**Platelets**	-0.03 (-0.62−0.23)	0.13 (-0.35−0.65)	0.452
**Protein synthesis (I)**	-0.78 (-1.17−0.29)	-0.35 (-0.91−0.13)	0.667
**Regulation of antigen presentation and immune response**	-1.41 (-1.43−-0.86)	-0.73 (-1.26−-0.22)	0.334
**Regulation of transcription, transcription factors**	-0.82 (-0.92−-0.17)	-0.45 (-0.81−-0.10)	0.629

Data are presented as median (interquartile range). The total number of patients with available data is indicated. All *p* values are derived from non-parametrical Mann-Whitney *U* tests. Statistically significant *p* values are in bold.

βGPI: $β_2$ glycoprotein I; CORO1A-DEF6: coronin 1A-differentially expressed in FDCP 6 homolog; Ig: immunoglobulin; LN: lupus nephritis; MAPK: mitogen-activated protein kinase.

Supplementary Table S25. Z-scores of replicated gene modules in patients with LN and aCL IgG positivity versus negative patients

Gene module	aCL IgG (+) N=4	aCL IgG (-) N=27	*p* value
**B cell**	-1.51 (-1.97−-1.05)	-1.71 (-2.36−-0.43)	0.906
**Cell cycle, mitotic phase**	-0.15 (-0.56−0.16)	-0.43 (-0.87−-0.13)	0.480
**CORO1A-DEF6 network**	-0.21 (-0.45−-0.04)	-0.09 (-0.20−0.02)	0.289
**Enriched in antigen presentation (III)**	-0.48 (-0.88−-0.32)	-0.37 (-0.51−0.03)	0.289
**Enriched in neutrophils (II)**	0.02 (-0.25−0.19)	0.08 (-0.07−0.18)	0.680
**Erythrocytes**	-0.02 (-0.25−0.41)	0.21 (-0.49−0.65)	0.953
**Extracellular matrix (I)**	-1.28 (-1.61−-0.97)	-0.74 (-1.15−-0.36)	0.157
**Extracellular region cluster**	-0.20 (-0.47−0.03)	-0.08 (-0.25−0.02)	0.480
**Inflammation (IV)**	0.37 (0.16−0.70)	0.18 (-0.07−0.43)	0.377
**Inflammation (VI)**	-0.21 (-0.66−0.17)	0.02 (-0.24−0.22)	0.517
**Inositol phosphate metabolism**	0.28 (-0.23−0.78)	-0.07 (-0.39−0.25)	0.377
**Interferon**	0.39 (-0.01−0.99)	1.49 (0.05−2.12)	0.289
**Interferon (II)**	-0.02 (-0.29−0.41)	0.54 (0.24−0.73)	0.157
**MAPK−RAS signalling**	-0.15 (-0.78−0.40)	0.02 (-0.41−0.33)	0.768
**Monocytes**	0.32 (0.10−0.75)	0.26 (-0.10−0.92)	0.596
**Plasma cells, immunoglobulins**	-0.10 (-0.38−0.29)	-0.10 (-0.67−0.65)	0.953
**Platelets**	0.10 (-0.18−0.42)	0.01 (-0.37−0.63)	0.953
**Protein synthesis (I)**	-0.24 (-0.90−0.32)	-0.44 (-0.93−0.10)	0.860
**Regulation of antigen presentation and immune response**	-1.14 (-1.61−-0.63)	-0.80 (-1.35−-0.25)	0.517
**Regulation of transcription, transcription factors**	-0.50 (-0.85−-0.07)	-0.46 (-0.91−-0.13)	0.724

Data are presented as median (interquartile range). The total number of patients with available data is indicated. All *p* values are derived from non-parametrical Mann-Whitney *U* tests. Statistically significant *p* values are in bold.

aCL: anti-cardiolipin; CORO1A-DEF6: coronin 1A- differentially expressed in FDCP 6 homolog; Ig: immunoglobulin; LN: lupus nephritis; MAPK: mitogen-activated protein kinase.

Supplementary Table S26. Z-scores of replicated gene modules in patients with LN and low versus normal/high levels of C3c

Gene module	Low C3c<br>N=10	Normal/high C3c<br>N=21	*p* value
**B cell**	-1.03 (-2.54−-0.32)	-1.71 (-2.20−-0.68)	0.642
**Cell cycle, mitotic phase**	-0.47 (-0.88−0.38)	-0.32 (-0.76−-0.12)	0.899
**CORO1A-DEF6 network**	-0.07 (-0.18−0.10)	-0.12 (-0.28−0.00)	0.422
**Enriched in antigen presentation (III)**	-0.06 (-0.32−0.17)	-0.43 (-0.59−-0.24)	**0.031**
**Enriched in neutrophils (II)**	0.17 (0.08−0.20)	-0.03 (-0.14−0.14)	**0.010**
**Erythrocytes**	-0.24 (-0.50−-0.16)	0.33 (-0.23−0.72)	0.083
**Extracellular matrix (I)**	-0.52 (-0.79−-0.13)	-1.00 (-1.26−-0.58)	0.083
**Extracellular region cluster**	-0.01 (-0.12−0.13)	-0.10 (-0.27−-0.02)	0.099
**Inflammation (IV)**	-0.12 (-0.34−0.17)	0.33 (0.07−0.50)	**0.022**
**Inflammation (VI)**	0.23 (0.06−0.36)	-0.19 (-0.30−0.11)	**0.004**
**Inositol phosphate metabolism**	-0.25 (-0.57−0.36)	-0.03 (-0.28−0.28)	0.375
**Interferon**	1.73 (0.37−2.26)	0.76 (-0.06−1.74)	0.099
**Interferon (II)**	0.73 (0.38−0.98)	0.44 (-0.09−0.60)	**0.025**
**MAPK−RAS signalling**	0.29 (-0.13−0.33)	-0.35 (-0.68−0.36)	0.151
**Monocytes**	0.56 (0.11−1.06)	0.26 (-0.46−0.44)	0.353
**Plasma cells, immunoglobulins**	0.39 (-0.32−0.81)	-0.23 (-0.81−0.39)	0.205
**Platelets**	-0.21 (-0.50−0.19)	0.25 (-0.24−0.68)	0.083
**Protein synthesis (I)**	-0.73 (-1.07−0.11)	-0.18 (-0.90−0.16)	0.526
**Regulation of antigen presentation and immune response**	-0.99 (-1.16−-0.14)	-0.80 (-1.62−-0.29)	0.583
**Regulation of transcription, transcription factors**	-0.47 (-0.96−-0.09)	-0.46 (-0.82−-0.20)	0.833

Data are presented as median (interquartile range). The total number of patients with available data is indicated. All *p* values are derived from non-parametrical Mann-Whitney *U* tests. Statistically significant *p* values are in bold.

C3c: complement component 3c; CORO1A-DEF6: coronin 1A- differentially expressed in FDCP 6 homolog; Ig: immunoglobulin; LN: lupus nephritis; MAPK: mitogen-activated protein kinase.

Supplementary Table S27. Z-scores of replicated gene modules in patients with LN and low versus normal/high levels of C4

Gene module	Low C4<br>N=9	Normal/high C4<br>N=22	*p* value
**B cell**	-1.22 (-2.20−-0.91)	-1.76 (-2.55−-0.15)	0.761
**Cell cycle, mitotic phase**	-0.26 (-0.48−-0.06)	-0.44 (-0.98−-0.15)	0.240
**CORO1A-DEF6 network**	-0.01 (-0.17−0.07)	-0.11 (-0.29−0.00)	0.277
**Enriched in antigen presentation (III)**	-0.15 (-0.86−0.17)	-0.39 (-0.53−-0.15)	0.663
**Enriched in neutrophils (II)**	0.09 (0.00−0.19)	0.04 (-0.12−0.18)	0.572
**Erythrocytes**	-0.15 (-0.30−0.29)	0.20 (-0.42−0.71)	0.408
**Extracellular matrix (I)**	-0.55 (-0.85−-0.23)	-0.97 (-1.37−-0.51)	0.164
**Extracellular region cluster**	-0.08 (-0.14−0.03)	-0.09 (-0.28−0.02)	0.542
**Inflammation (IV)**	0.34 (-0.14−0.40)	0.21 (-0.01−0.48)	0.728
**Inflammation (VI)**	0.11 (0.01−0.36)	-0.04 (-0.27−0.18)	0.164
**Inositol phosphate metabolism**	0.05 (-0.36−0.45)	-0.08 (-0.40−0.19)	0.408
**Interferon**	1.71 (0.76−2.10)	0.88 (0.00−1.78)	0.408
**Interferon (II)**	0.60 (0.12−0.70)	0.45 (0.23−0.73)	0.632
**MAPK−RAS signalling**	-0.26 (-0.46−0.16)	0.16 (-0.47−0.37)	0.177
**Monocytes**	0.29 (0.25−1.09)	0.23 (-0.44−0.66)	0.240
**Plasma cells, immunoglobulins**	0.16 (-0.34−0.78)	-0.23 (-0.76−0.41)	0.317
**Platelets**	-0.18 (-0.53−0.28)	0.12 (-0.32−0.65)	0.486
**Protein synthesis (I)**	-0.18 (-0.46−0.31)	-0.55 (-1.00−0.03)	0.139
**Regulation of antigen presentation and immune response**	-0.85 (-1.15−0.02)	-0.83 (-1.65−-0.33)	0.296
**Regulation of transcription, transcription factors**	0.17 (-0.67−0.22)	-0.56 (-0.95−-0.35)	0.033

Data are presented as median (interquartile range). The total number of patients with available data is indicated. All *p* values are derived from non-parametrical Mann-Whitney *U* tests. Statistically significant *p* values are in bold.

C4: complement component 4; CORO1A-DEF6: coronin 1A- differentially expressed in FDCP 6 homolog; Ig: immunoglobulin; LN: lupus nephritis; MAPK: mitogen-activated protein kinase.

Supplementary Table S28. Z-scores of replicated gene modules in patients with LN and anti-chromatin positivity versus negative patients

Gene module	Anti-chromatin (+) N=8	Anti-chromatin (-) N=22	*p* value
**B cell**	-1.76 (-2.54−-0.71)	-1.35 (-2.37−-0.32)	0.743
**Cell cycle, mitotic phase**	-0.24 (-1.05−-0.06)	-0.46 (-0.84−-0.15)	0.963
**CORO1A-DEF6 network**	0.00 (-0.20−0.01)	-0.11 (-0.22−0.01)	0.574
**Enriched in antigen presentation (III)**	-0.43 (-0.54−-0.17)	-0.39 (-0.55−0.03)	0.851
**Enriched in neutrophils (II)**	-0.07 (-0.15−0.16)	0.09 (0.01−0.18)	0.373
**Erythrocytes**	0.62 (-0.28−0.80)	0.02 (-0.43−0.52)	0.260
**Extracellular matrix (I)**	-0.83 (-1.07−-0.64)	-0.69 (-1.35−-0.40)	0.815
**Extracellular region cluster**	-0.10 (-0.26−-0.01)	-0.09 (-0.28−0.02)	0.925
**Inflammation (IV)**	0.11 (0.00−0.30)	0.27 (-0.13−0.49)	0.511
**Inflammation (VI)**	-0.16 (-0.27−-0.01)	0.07 (-0.17−0.25)	0.205
**Inositol phosphate metabolism**	-0.17 (-0.39−0.16)	-0.06 (-0.39−0.39)	0.673
**Interferon**	0.88 (0.13−1.57)	1.70 (0.05−2.13)	0.606
**Interferon (II)**	0.41 (0.24−0.60)	0.57 (0.16−0.82)	0.511
**MAPK−RAS signalling**	-0.02 (-0.64−0.41)	0.02 (-0.33−0.33)	0.925
**Monocytes**	0.23 (-0.47−0.62)	0.28 (0.01−0.83)	0.851
**Plasma cells, immunoglobulins**	-0.49 (-0.93−0.02)	0.10 (-0.55−0.76)	0.133
**Platelets**	0.11 (-0.26−0.40)	-0.01 (-0.50−0.65)	0.606
**Protein synthesis (I)**	-0.35 (-0.93−-0.09)	-0.45 (-0.93−0.17)	0.888
**Regulation of antigen presentation and immune response**	-0.64 (-1.37−-0.38)	-0.96 (-1.42−-0.37)	0.778
**Regulation of transcription, transcription factors**	-0.45 (-0.73−-0.34)	-0.43 (-0.84−0.07)	0.673

Data are presented as median (interquartile range). The total number of patients with available data is indicated. All *p* values are derived from non-parametrical Mann-Whitney *U* tests. Statistically significant *p* values are in bold.

CORO1A-DEF6: coronin 1A- differentially expressed in FDCP 6 homolog; Ig: immunoglobulin; LN: lupus nephritis; MAPK: mitogen-activated protein kinase.

Supplementary Table S29. Z-scores of replicated gene modules in patients with LN and anti-dsDNA positivity versus negative patients

Gene module	anti-dsDNA (+) N=18	anti-dsDNA (-) N=13	*p* value
**B cell**	-1.03 (-2.30−-0.05)	-1.81 (-3.26−-1.48)	0.161
**Cell cycle, mitotic phase**	-0.41 (-0.69−-0.12)	-0.32 (-1.16−-0.06)	0.471
**CORO1A-DEF6 network**	-0.05 (-0.18−0.03)	-0.13 (-0.30−0.00)	0.401
**Enriched in antigen presentation (III)**	-0.16 (-0.45−0.14)	-0.44 (-0.56−-0.28)	0.093
**Enriched in neutrophils (II)**	0.09 (0.02−0.18)	-0.05 (-0.14−0.18)	0.337
**Erythrocytes**	-0.16 (-0.50−0.32)	0.57 (-0.23−0.79)	0.139
**Extracellular matrix (I)**	-0.52 (-1.12−-0.25)	-1.00 (-1.26−-0.74)	0.078
**Extracellular region cluster**	-0.05 (-0.15−0.03)	-0.14 (-0.40−-0.02)	0.186
**Inflammation (IV)**	0.24 (-0.18−0.44)	0.25 (0.06−0.49)	0.575
**Inflammation (VI)**	0.07 (0.00−0.24)	-0.25 (-0.30−0.20)	0.078
**Inositol phosphate metabolism**	-0.03 (-0.34−0.39)	-0.07 (-0.40−0.21)	0.936
**Interferon**	1.72 (0.05−2.22)	0.76 (-0.01−1.69)	0.246
**Interferon (II)**	0.62 (0.23−0.82)	0.44 (0.13−0.60)	0.186
**MAPK−RAS signalling**	0.09 (-0.28−0.33)	-0.37 (-0.68−0.37)	0.423
**Monocytes**	0.34 (0.04−0.95)	0.26 (-0.49−0.44)	0.471
**Plasma cells, immunoglobulins**	0.03 (-0.49−0.74)	-0.37 (-0.91−0.42)	0.262
**Platelets**	-0.10 (-0.39−0.51)	0.25 (-0.24−0.67)	0.378
**Protein synthesis (I)**	-0.45 (-0.88−0.11)	-0.13 (-1.03−0.17)	0.936
**Regulation of antigen presentation and immune response**	-0.85 (-1.16−-0.08)	-0.80 (-1.62−-0.44)	0.471
**Regulation of transcription, transcription factors**	-0.51 (-0.93−0.07)	-0.46 (-0.82−-0.35)	0.779

Data are presented as median (interquartile range). The total number of patients with available data is indicated. All *p* values are derived from non-parametrical Mann-Whitney *U* tests. Statistically significant *p* values are in bold.

CORO1A-DEF6: coronin 1A- differentially expressed in FDCP 6 homolog; Ig: immunoglobulin; LN: lupus nephritis; MAPK: mitogen-activated protein kinase.

Supplementary Table S30. Z-scores of replicated gene modules in patients with LN and anti-SSA-52 positivity versus negative patients

Gene module	anti-SSA-52 (+) N=5	anti-SSA-52 (-) N=19	*p* value
**B cell**	-2.33 (-3.17−-1.22)	-1.48 (-2.06−-0.43)	0.303
**Cell cycle, mitotic phase**	-0.76 (-1.15−-0.51)	-0.32 (-0.87−0.05)	0.126
**CORO1A-DEF6 network**	-0.13 (-0.17−-0.12)	-0.04 (-0.34−0.03)	0.546
**Enriched in antigen presentation (III)**	-0.45 (-0.59−-0.44)	-0.28 (-0.55−0.03)	0.374
**Enriched in neutrophils (II)**	0.08 (0.00−0.23)	0.09 (-0.10−0.18)	0.696
**Erythrocytes**	0.19 (-0.15−0.80)	-0.17 (-0.49−0.48)	0.271
**Extracellular matrix (I)**	-0.85 (-1.15−-0.58)	-0.74 (-1.21−-0.36)	0.499
**Extracellular region cluster**	-0.15 (-0.42−-0.08)	-0.09 (-0.27−0.06)	0.241
**Inflammation (IV)**	0.25 (-0.14−0.50)	0.12 (-0.06−0.36)	0.749
**Inflammation (VI)**	0.25 (-0.22−0.36)	0.02 (-0.22−0.15)	0.414
**Inositol phosphate metabolism**	-0.39 (-0.55−-0.03)	-0.10 (-0.38−0.44)	0.271
**Interferon**	1.71 (1.33−2.10)	0.58 (0.01−1.73)	0.271
**Interferon (II)**	0.70 (0.54−0.93)	0.28 (0.02−0.66)	0.166
**MAPK−RAS signalling**	0.16 (0.03−0.28)	-0.22 (-0.66−0.35)	0.696
**Monocytes**	0.25 (-0.20−0.38)	0.26 (0.02−0.92)	0.546
**Plasma cells, immunoglobulins**	-0.34 (-0.73−0.61)	-0.10 (-0.58−0.80)	0.644
**Platelets**	0.28 (0.23−0.67)	-0.18 (-0.46−0.25)	0.145
**Protein synthesis (I)**	-0.68 (-1.12−-0.18)	-0.44 (-0.91−0.23)	0.303
**Regulation of antigen presentation and immune response**	-1.15 (-1.17−-1.06)	-0.80 (-1.35−-0.04)	0.214
**Regulation of transcription, transcription factors**	-0.35 (-0.82−-0.26)	-0.44 (-0.96−0.11)	0.915

Data are presented as median (interquartile range). The total number of patients with available data is indicated. All *p* values are derived from non-parametrical Mann-Whitney *U* tests. Statistically significant *p* values are in bold.

CORO1A-DEF6: coronin 1A- differentially expressed in FDCP 6 homolog; Ig: immunoglobulin; LN: lupus nephritis; MAPK: mitogen-activated protein kinase.

Supplementary Table S31. Z-scores of replicated gene modules in patients with LN and anti-SSA-60 positivity versus negative patients

Gene module	anti-SSA-60 (+) N=5	anti-SSA-60 (-) N=19	*p* value
**B cell**	-2.33 (-3.17−-1.54)	-1.16 (-2.06−-0.10)	0.095
**Cell cycle, mitotic phase**	-0.51 (-1.15−-0.26)	-0.43 (-0.87, -0.09)	0.594
**CORO1A-DEF6 network**	-0.17 (-0.18−-0.13)	-0.04 (-0.23−0.03)	0.241
**Enriched in antigen presentation (III)**	-0.45 (-0.59−-0.14)	-0.37 (-0.55−0.03)	0.644
**Enriched in neutrophils (II)**	0.08 (0.00−0.23)	0.09 (-0.10−0.18)	0.644
**Erythrocytes**	-0.15 (-0.23−0.19)	-0.13 (-0.49−0.70)	0.972
**Extracellular matrix (I)**	-1.15 (-1.15−-0.85)	-0.63 (-1.13−-0.36)	0.214
**Extracellular region cluster**	-0.08 (-0.15−-0.03)	-0.10 (-0.28−0.02)	0.972
**Inflammation (IV)**	0.25 (-0.14−0.49)	0.12 (-0.06−0.36)	0.915
**Inflammation (VI)**	0.25 (-0.22−0.36)	0.02 (-0.22−0.15)	0.455
**Inositol phosphate metabolism**	-0.39 (-0.55−0.28)	-0.10 (-0.38−0.32)	0.644
**Interferon**	1.71 (1.33−2.10)	0.58 (0.04−1.73)	0.546
**Interferon (II)**	0.70 (0.54−0.93)	0.28 (0.13−0.66)	0.303
**MAPK−RAS signalling**	0.16 (-0.46−0.28)	0.03 (-0.59−0.35)	0.972
**Monocytes**	0.38 (0.25−1.09)	0.21 (-0.18−0.65)	0.303
**Plasma cells, immunoglobulins**	-0.34 (-0.73−0.61)	-0.10 (-0.58−0.73)	0.804
**Platelets**	0.28 (0.23−1.02)	-0.18 (-0.46−0.25)	0.110
**Protein synthesis (I)**	-0.68 (-1.12−-0.18)	-0.44 (-0.91−0.10)	0.499
**Regulation of antigen presentation and immune response**	-1.15 (-1.17−-1.06)	-0.80 (-1.35−-0.13)	0.455
**Regulation of transcription, transcription factors**	-0.82 (-0.85−-0.26)	-0.39 (-0.84−0.11)	0.644

Data are presented as median (interquartile range). The total number of patients with available data is indicated. All *p* values are derived from non-parametrical Mann-Whitney *U* tests. Statistically significant *p* values are in bold.

CORO1A-DEF6: coronin 1A- differentially expressed in FDCP 6 homolog; Ig: immunoglobulin; LN: lupus nephritis; MAPK: mitogen-activated protein kinase.

Supplementary Table S32. Z-scores of replicated gene modules in patients with LN and anti-U1RNP positivity versus negative patients

Gene module	Anti-U1RNP (+) N=5	Anti-U1RNP (-) N=20	*p* value
**B cell**	0.01 (-0.65−0.03)	-1.84 (-2.40−-1.11)	0.089
**Cell cycle, mitotic phase**	-0.76 (-1.05−-0.48)	-0.29 (-0.87−-0.01)	0.197
**CORO1A-DEF6 network**	-0.04 (-0.09−0.02)	-0.13 (-0.33−0.02)	0.342
**Enriched in antigen presentation (III)**	0.03 (-0.44−0.20)	-0.41 (-0.75−-0.15)	0.174
**Enriched in neutrophils (II)**	0.02 (-0.14−0.17)	0.09 (-0.07−0.19)	0.541
**Erythrocytes**	0.19 (-0.13−0.72)	-0.16 (-0.49−0.60)	0.248
**Extracellular matrix (I)**	-0.48 (-0.58−-0.33)	-0.80 (-1.30−-0.52)	0.221
**Extracellular region cluster**	-0.29 (-0.42−-0.25)	-0.09 (-0.26−0.05)	0.118
**Inflammation (IV)**	0.25 (-0.50−0.29)	0.14 (-0.04−0.41)	0.587
**Inflammation (VI)**	0.07 (-0.14−0.20)	0.01 (-0.26−0.21)	0.634
**Inositol phosphate metabolism**	-0.28 (-0.55−-0.11)	0.07 (-0.39−0.43)	0.118
**Interferon**	0.58 (0.30−1.71)	1.41 (0.04−2.25)	0.587
**Interferon (II)**	0.24 (0.13−0.76)	0.53 (0.20−0.69)	0.839
**MAPK−RAS signalling**	0.52 (0.36−0.55)	-0.27 (-0.68−0.29)	0.007
**Monocytes**	0.38 (0.09−0.38)	0.23 (-0.24−1.01)	0.684
**Plasma cells, immunoglobulins**	-0.23 (-0.55−-0.10)	-0.10 (-0.77−0.79)	0.786
**Platelets**	0.23 (0.01−0.67)	-0.10 (-0.43−0.31)	0.248
**Protein synthesis (I)**	-0.26 (-1.27−-0.04)	-0.45 (-0.92−0.20)	0.415
**Regulation of antigen presentation and immune response**	-0.64 (-1.12−-0.29)	-0.96 (-1.41−-0.17)	0.839
**Regulation of transcription, transcription factors**	-0.82 (-1.10−-0.35)	-0.42 (-0.72−0.11)	0.277

Data are presented as median (interquartile range). The total number of patients with available data is indicated. All *p* values are derived from non-parametrical Mann-Whitney *U* tests. Statistically significant *p* values are in bold.

CORO1A-DEF6: coronin 1A- differentially expressed in FDCP 6 homolog; Ig: immunoglobulin; LN: lupus nephritis; MAPK: mitogen-activated protein kinase.

Supplementary Table S33. The most enriched signalling molecule networks in patients with LN

Regulator	Motif ID	AUC	NES	Targets	TFs
**Interferon gene module**					
STAT1	factorbook-STAT2	0.229	22.37	59	13
IRF8	taipale-NCGAAACCGAAACY-IRF8-DBD	0.207	20.12	57	8
STAT1	jaspar-MA0137.1	0.206	19.98	65	12
**Interferon (II) gene module**					
1RF9	taipale-AWCGAAACCGAAACY-IRF9-full	0.147	13.16	48	6
IRF8	taipale-NCGAAACCGAAACT-IRF8-full	0.140	12.47	47	6
IRF8	taipale-NCGAAACCGAAACY-IRF8-DBD	0.136	12.03	24	8
**B cell gene module**					
SOX10	tfdimers-MD00329	0.101	4.78	12	2
PRDM1	flyfactorsurvey-Blimp-1_SOLEXA_FBgn0035625	0.096	4.42	24	14
TBX2	taipale-NAGGTGTGAWN-TBX2-full	0.095	4.36	11	12
**Plasma cells, immunoglobulins gene module**					
TFDP3	transfac_pro-M00920	0.079	8.38	65	8
NFYC	yetfasco-1537	0.077	8.03	96	4
NFYC	jaspar-MA0316.1	0.076	7.91	95	4
**Regulation of transcription, transcription factors**					
YY1	flyfactorsurvey-phol_SOLEXA_5_FBgn0035997	0.162	8.83	18	3
YY1	flyfactorsurvey-phol_SANGER_5_FBgn0035997	0.149	8.04	9	2
YY1	factorbook-YY1	0.143	7.73	19	3
**Cell cycle, mitotic phase gene module**					
YY1	jaspar-PF0063.1	0.059	4.50	25	1
NANOS1	hdpi-NANOS1	0.058	4.40	9	1
YY1	flyfactorsurvey-phol_SOLEXA_5_FBgn0035997	0.057	4.32	39	3
**Protein synthesis (I) gene module**					
ZBTB33	jaspar-PF0008.1	0.030	3.91	143	1
ZBTB33	factorbook-UA1	0.029	3.56	108	1
ZBTB33	homer-M00076	0.029	3.54	135	1
**Extracellular matrix (I) gene module**					
NRF1	factorbook-NRF1	0.037	5.32	202	2
NRF1	swissregulon-NRF1.p2	0.036	5.09	131	2
ZSCAN10	jaspar-PF0001.1	0.036	4.91	191	3
**Regulation of antigen presentation and immune response gene module**					
PRDM2	yetfasco-1185	0.027	4.29	562	9
ZNF143	jaspar-PF0113.1	0.027	4.15	82	1
CUX1	homer-M00467	0.027	4.09	90	2

Genes in replicated gene modules with a mean |z-score| >1 in at least one LN patient subgroup were imputed in iRegulon through Cytoscape to generate signalling molecule networks and identify their chief regulators. The top chief regulators and enriched motifs are displayed.

AUC: area under the curve; LN: lupus nephritis; NES: normalised enrichment score; TFs: transcription factors.

Supplementary Figure S1. The *NRF1* signalling molecule network and annotated drug targets in patients with lupus nephritis

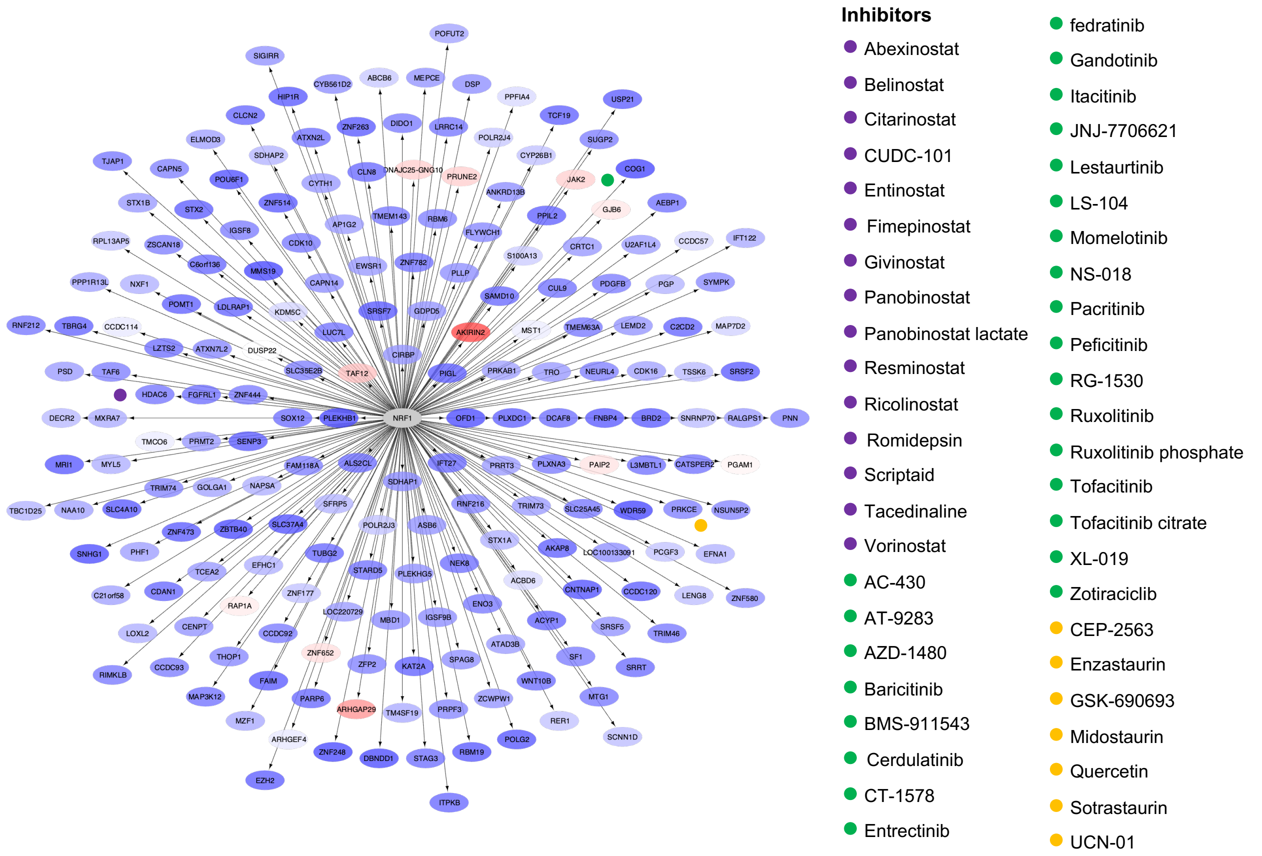

Genes in the extracellular matrix (I) gene module were imputed in iRegulon through Cytoscape to generate signalling molecule networks and identify their chief regulators. One of the most enriched signalling molecule networks is plotted, with the chief regulator *NRF1* in the central node. The colour of the nodes ranges from light blue (downregulated genes) to increasing intensities of red (upregulated genes) based on the gene dysregulation (z-scores) in the lo-IFN patient subgroup. Inhibiting drugs and their upregulated targets are indicated by coloured dots.

IFN: interferon; lo; low.

Supplementary Figure S2. The *YY1* signalling molecule network and annotated drug targets in patients with lupus nephritis

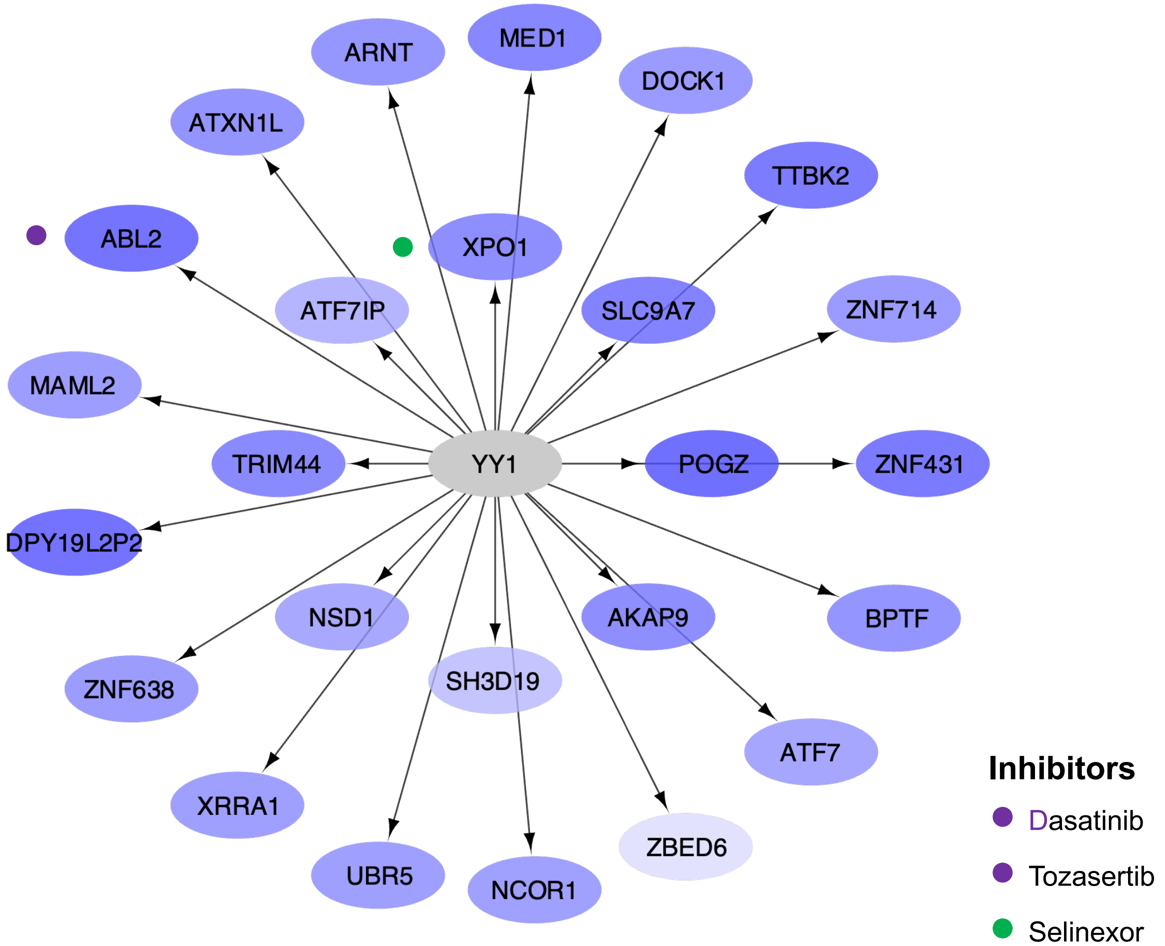

Genes in the cell cycle, mitotic phase gene module were imputed in iRegulon through Cytoscape to generate signalling molecule networks and identify their chief regulators. One of the most enriched signalling molecule networks is plotted, with the chief regulator *YY1* in the central node. The colour of the nodes ranges from light blue (downregulated genes) to increasing intensities of red (upregulated genes) based on the gene dysregulation (z-scores) in the lo-IFN patient subgroup. Inhibiting drugs and their upregulated targets are indicated by coloured dots.

IFN: interferon; lo; low.

Supplementary Figure S3. The *ZBTB33* signalling molecule network and annotated drug targets in patients with lupus nephritis

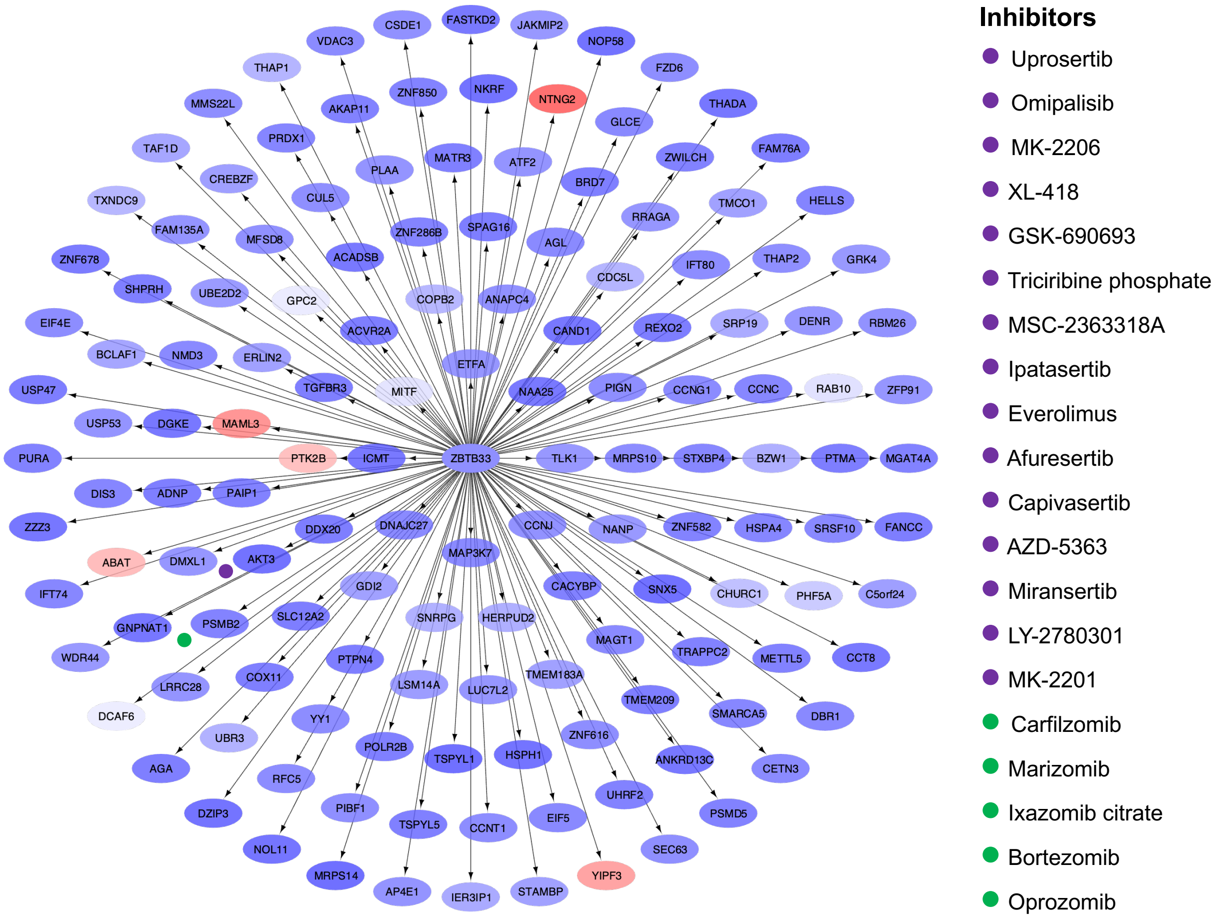

Genes in the protein synthesis (I) gene module were imputed in iRegulon through Cytoscape to generate signalling molecule networks and identify their chief regulators. One of the most enriched signalling molecule networks is plotted, with the chief regulator *ZBTB33* in the central node. The colour of the nodes ranges from light blue (downregulated genes) to increasing intensities of red (upregulated genes) based on the gene dysregulation (z-scores) in the lo-IFN patient subgroup. Inhibiting drugs and their upregulated targets are indicated by coloured dots.

IFN: interferon; lo; low.

Supplementary Figure S4. The *PRDM2* signalling molecule network and annotated drug targets in patients with lupus nephritis

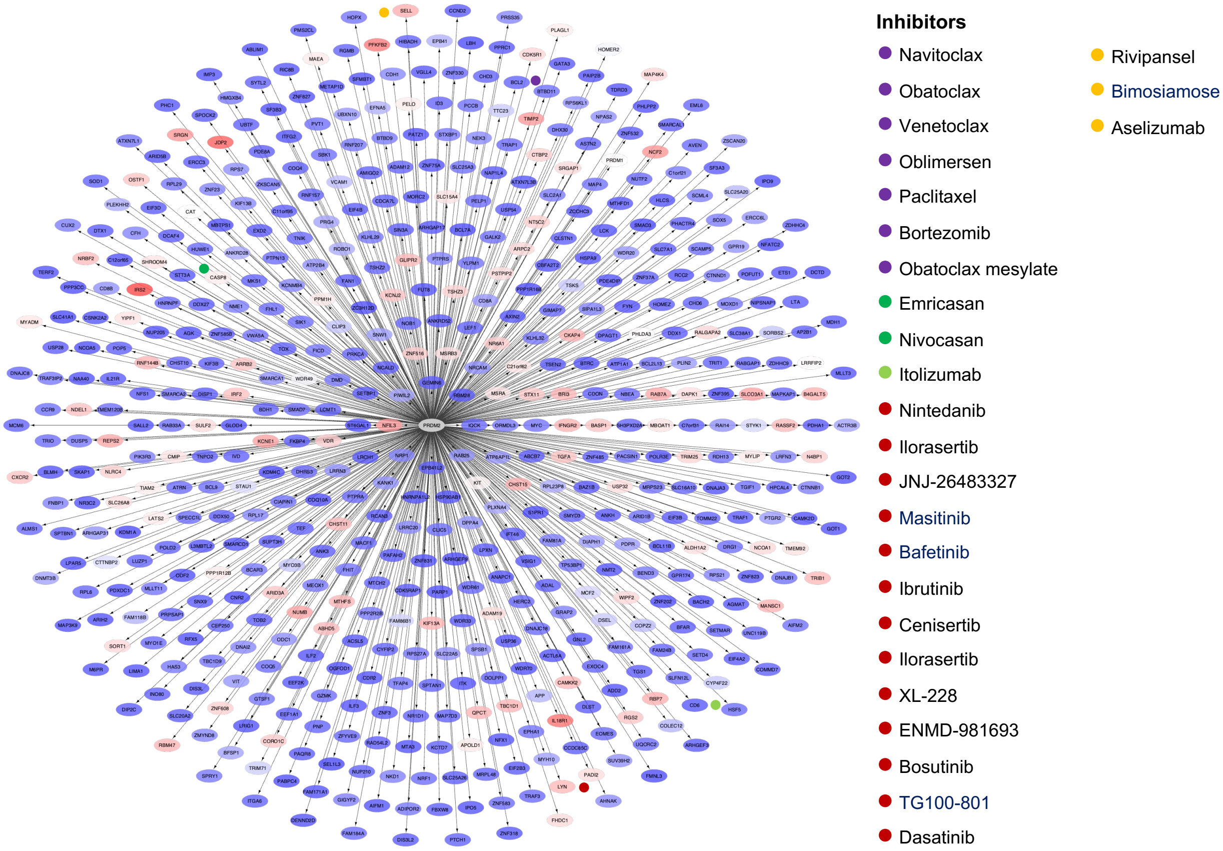

Genes in the regulation of transcription, transcription factors gene module were imputed in iRegulon through Cytoscape to generate signalling molecule networks and identify their chief regulators. One of the most enriched signalling molecule networks is plotted, with the chief regulator *PRDM2* in the central node. The colour of the nodes ranges from light blue (downregulated genes) to increasing intensities of red (upregulated genes) based on the gene dysregulation (z-scores) in the lo-IFN patient subgroup. Inhibiting drugs and their upregulated targets are indicated by coloured dots.

IFN: interferon; lo; low.

Supplementary Table S34. Response scores to selected targets in patients with LN

Target	lo-IFN subgroup<br><br>N=13	im-IFN subgroup<br><br>N=9	hi-IFN subgroup<br><br>N=19	lo-IFN vs im-IFN *p* value	lo-IFN vs hi-IFN *p* value	im-IFN vs hi-IFN *p* value
**IFNAR**	0.58□ 0.05	0.55□0.03	0.60□0.05	0.133	0.309	**0.015**
**CD22**	0.07□0.01	0.08□0.00	0.08□0.01	0.077	**0.027**	0.539
**BAFF**	0.25□0.02	0.24□0.02	0.25□0.03	0.171	0.803	0.337
**BAFF and APRIL**	0.25□0.02	0.24□0.02	0.25□0.03	0.171	0.803	0.337
**BAFFR**	0.03□0.01	0.04□0.01	0.03□0.01	**0.010**	0.309	0.052
**Calcineurin**	0.07□0.01	0.05□0.01	0.05□0.01	**0.001**	**<0.001**	0.446
**C5**	0.01□0.00	0.01□0.00	0.01□0.00	0.217	0.454	0.363
**CD19**	0.18□0.08	0.26□0.03	0.22□0.04	**0.005**	0.328	**0.010**
**CD38**	0.36□0.02	0.40□0.05	0.40□0.03	**0.021**	**<0.001**	0.476
**mTORC1**	0.76□0.04	0.71□0.03	0.72□0.04	**0.001**	**0.003**	0.337
**CCR1**	0.00□0.00	0.00□0.00	0.00□0.01	N/A	0.408	0.491
**BTK**	0.14□0.02	0.17□0.01	0.16□0.01	**0.001**	**0.001**	**0.029**
**Tyrosine kinase**	0.81□0.04	0.75□0.02	0.76□0.05	**<0.001**	**0.016**	0.313
**JAK1/JAK2**	0.96□0.04	0.89□0.03	0.93□0.05	**0.001**	0.388	**0.022**

Data are presented as mean (standard deviation). All *p* values are derived from non-parametrical Mann-Whitney *U* tests. Statistically significant *p* values are in bold.

APRIL: a proliferation-inducing ligand; BAFF: B cell activating factor belonging to the tumour necrosis factor family; BAFFR; B cell activating factor belonging to the tumour necrosis factor family; BTK: Bruton's tyrosine kinase; C5: complement component 5; hi: high; IFN: interferon; IFNAR: interferon-α/β receptor; im: intermediate; N/A: not applicable; JAK: Janus kinase; LN: lupus nephritis; lo: low; mTORC1: mammalian target of rapamycin complex 1.

Supplementary Table S35. LN patients with anticipated response to selected drug targets

Target	lo-IFN subgroup	im-IFN subgroup	hi-IFN subgroup	lo-IFN vs im-IFN	lo-IFN vs hi-IFN	im-IFN vs hi-IFN
	**N=13**	**N=9**	**N=19**	***p* value**	***p* value**	***p* value**
**IFNAR**	5 (38.5%)	1 (11.1%)	14 (73.7%)	0.333	0.104	**0.004**
**CD22**	4 (30.8%)	6 (66.7%)	14 (73.7%)	0.192	**0.041**	1.000
**BAFF**	7 (53.8%)	3 (33.3%)	10 (52.6%)	0.415	1.000	0.435
**BAFF and APRIL**	7 (53.8%)	3 (33.3%)	10 (52.6%)	0.415	1.000	0.435
**BAFFR**	6 (46.2%)	8 (88.9%)	12 (63.2%)	0.074	0.556	0.214
**Calcineurin**	12 (92.3%)	2 (22.2%)	5 (26.3%)	**0.001**	**0.001**	1.000
**C5**	4 (30.8%)	6 (66.7%)	10 (52.6%)	0.192	0.389	0.687
**CD19**	5 (38.5%)	8 (88.9%)	8 (42.1%)	**0.031**	1.000	**0.039**
**CD38**	0 (0.0%)	4 (44.4%)	13 (68.4%)	**0.017**	**<0.001**	0.409
**mTORC1**	10 (76.9%)	1 (11.1%)	5 (26.3%)	**0.008**	**0.014**	0.630
**CCR1**	0 (0.0%)	0 (0.0%)	1 (5.3%)	1.000	1.000	1.000
**BTK**	1 (7.7%)	9 (100.0%)	12 (63.2%)	**<0.001**	**0.003**	0.062
**Tyrosine kinase**	12 (92.3%)	1 (11.1%)	6 (31.6%)	**<0.001**	**0.002**	0.371
**JAK1/JAK2**	10 (76.9%)	0 (0.0%)	12 (63.2%)	**<0.001**	0.467	**0.003**

Data are presented as number (percentage). All *p* values are derived from Pearson's chi squared ($\chi 2$) or Fisher's exact tests. Statistically significant *p* values are in bold.

APRIL: a proliferation-inducing ligand; BAFF: B cell activating factor belonging to the tumour necrosis factor family; BAFFR; B cell activating factor belonging to the tumour necrosis factor family; BTK: Bruton's tyrosine kinase; C5: complement component 5; hi: high; IFN: interferon; IFNAR: interferon-α/β receptor; im: intermediate; JAK: Janus kinase; LN: lupus nephritis; lo: low; mTORC1: mammalian target of rapamycin complex 1.
